# AI-Driven Thoracic X-ray Diagnostics: Transformative Transfer Learning for Clinical Validation in Pulmonary Radiography

**DOI:** 10.3390/jpm14080856

**Published:** 2024-08-12

**Authors:** Md Abu Sufian, Wahiba Hamzi, Tazkera Sharifi, Sadia Zaman, Lujain Alsadder, Esther Lee, Amir Hakim, Boumediene Hamzi

**Affiliations:** 1 IVR Low-Carbon Research Institute, Chang’an University, Xi’an 710018, China; 2017901635@chd.edu.cn; 2 School of Computing and Mathematical Sciences, University of Leicester, Leicester LE1 7RH, UK; 3 Laboratoire de Biotechnologie Santé et Environnement, Department of Biology, University of Blida, Blida 09000, Algeria; 4 Data Science Architect-Lead Technologist, Booz Allen Hamilton, Texas City, TX 78226, USA; 5 Department of Physiology, Queen Mary University, London E1 4NS, UK; 6 Department of Computing and Mathematical Sciences, California Institute of Technology, Caltech, CA 91125, USA; 7 The Alan Turing Institute, London NW1 2DB, UK; 8 Department of Mathematics, Imperial College London, London SW7 2AZ, UK; 9 Department of Mathematics, Gulf University for Science and Technology (GUST), Mubarak Al-Abdullah 32093, Kuwait

**Keywords:** artificial intelligence, deep learning, diagnostic accuracy, medical imaging, model interpretability, pulmonary radiography

## Abstract

Our research evaluates advanced artificial (AI) methodologies to enhance diagnostic accuracy in pulmonary radiography. Utilizing DenseNet121 and ResNet50, we analyzed 108,948 chest X-ray images from 32,717 patients and DenseNet121 achieved an area under the curve (AUC) of 94% in identifying the conditions of pneumothorax and oedema. The model’s performance surpassed that of expert radiologists, though further improvements are necessary for diagnosing complex conditions such as emphysema, effusion, and hernia. Clinical validation integrating Latent Dirichlet Allocation (LDA) and Named Entity Recognition (NER) demonstrated the potential of natural language processing (NLP) in clinical workflows. The NER system achieved a precision of 92% and a recall of 88%. Sentiment analysis using DistilBERT provided a nuanced understanding of clinical notes, which is essential for refining diagnostic decisions. XGBoost and SHapley Additive exPlanations (SHAP) enhanced feature extraction and model interpretability. Local Interpretable Model-agnostic Explanations (LIME) and occlusion sensitivity analysis further enriched transparency, enabling healthcare providers to trust AI predictions. These AI techniques reduced processing times by 60% and annotation errors by 75%, setting a new benchmark for efficiency in thoracic diagnostics. The research explored the transformative potential of AI in medical imaging, advancing traditional diagnostics and accelerating medical evaluations in clinical settings.

## 1. Introduction

Despite advancements in artificial intelligence (AI) for medical imaging, challenges remain in diagnosing early stages of pulmonary diseases with high accuracy. This study aims to leverage advanced deep-learning techniques to improve diagnostic precision. In the advanced landscape of medical technology, AI has emerged as a game-changer. This project focuses on one specific and vital application: diagnosing diseases from chest X-ray images. We aim to build a classifier that is a machine learning model-based state-of-the-art solution—meaning it performs at or near the best achievable accuracy for this task. Chest X-ray is a widely used medical imaging modality for diagnosing various diseases, including pneumonia, tuberculosis, and lung cancer. However, manual interpretation of chest X-rays is challenging and time-consuming, especially for radiologists who need to read many images per day [1]. The motivation behind using AI for chest X-ray diagnostics stems from several critical factors. First, the increasing global burden of respiratory diseases underscores the need for efficient diagnostic tools. According to the World Health Organization (WHO), respiratory diseases account for a significant portion of global morbidity and mortality, with conditions like pneumonia, tuberculosis, and lung cancer being major contributors [2]. Early and accurate diagnosis is crucial for effective treatment and improved patient outcomes. Second, there is a substantial shortage of radiologists worldwide, particularly in low-resource settings. In many parts of the world, access to expert radiological services is limited, leading to delays in diagnosis and treatment. AI-driven solutions have the potential to bridge this gap by providing high-quality diagnostic services irrespective of geographical constraints. Deep learning, a subset of AI, offers a promising approach to automating and enhancing the accuracy of chest X-ray diagnosis. Deep learning algorithms, particularly convolutional neural networks (CNNs) trained to learn complex patterns in chest X-ray images and identify abnormalities with high sensitivity and specificity. The application of deep learning in medical imaging has shown significant promise, with numerous studies demonstrating its potential to match or even surpass human performance in certain diagnostic tasks [3]. Explainable AI (XAI) methods, such as SHapley Additive exPlanations (SHAP) and Local Interpretable Model-agnostic Explanations (LIME), play a critical role in medical imaging. These techniques provide insights into the model’s decision-making process, making the predictions more understandable and trustworthy for clinicians. We emphasized the significance of SHAP and LIME in validating our AI models and ensuring their clinical applicability.

### 1.1. Aim & Objectives

Our research project aims to develop a state-of-the-art chest X-ray medical diagnosis system using deep learning. The objectives include enhancing model accuracy and interpretability using techniques like LIME, SHAP, and Grad-CAM. This research seeks to contribute to the field of medical imaging by providing a robust diagnostic tool for COVID-19.

### 1.2. Novelty and Contributions

The research study impacted on several novel contributions to the field of medical imaging and deep learning for disease detection, with a particular focus on thoracic diseases including COVID-19. The research stands out due to its utilization of a comprehensive dataset consisting of 108,948 images from 32,717 patients, which ensures robust and generalizable results. This extensive dataset allows for a thorough analysis and validation of the models across a diverse set of conditions and patient demographics.

A key novelty of this work is the integration of multiple advanced models and techniques, such as DenseNet121, ResNet50, Grad-CAM, SHAP, LIME, VGG19, LDA, NER, Sentiment Analysis, BERT, XGBoost, and Occlusion Sensitivity. This combination enhances both the accuracy and interpretability of the diagnostic tool, leveraging the strengths of various deep learning approaches. The models achieved impressive performance metrics, including an AUC of 94%, precision of 92%, and recall of 88%, demonstrating their effectiveness in accurately diagnosing thoracic diseases.

Furthermore, the study includes real-world clinical validation, showing that AI can reduce processing times by 60% and annotation errors by 75%. This real-world application explored the practical relevance and potential impact of the models in clinical settings. By addressing critical challenges such as bias, computational expense, and interpretability, the research ensured that the models are not only accurate but also efficient and transparent.

Despite these achievements, the study identifies the need for further clinical validation and broader deployment, highlighting the ongoing research gap and setting the stage for future studies. Highlighting these contributions significantly advance the current state of automated disease detection using deep learning, providing practical tools and insights for improving diagnostic accuracy, efficiency, and interpretability in medical imaging.

### 1.3. Significance of the Study

The successful development of this system could significantly impact how we diagnose and treat diseases. Automating the interpretation of chest X-rays could help radiologists be more efficient and accurate, and it could also make chest X-ray diagnosis more accessible to patients in underserved areas. In addition to the potential clinical benefits, this project could also contribute to advancing deep learning for medical image analysis. The system has developed using various cutting-edge deep learning techniques, and the findings of this project could improve the performance of deep learning algorithms for other medical imaging tasks. Our research project has the potential to significantly contribute to the field of chest X-ray medical diagnosis and the advancement of deep learning for medical image analysis. Integrating AI into diagnostic processes represents a step forward in medical technology, offering a pathway to more efficient, accurate, and accessible healthcare solutions.

## 2. Methodology

### 2.1. Dataset Overview

The dataset used in our research is derived from the NIH ChestX-ray8 dataset, which is publicly available and compiled by the National Institute of Health Clinical Center. In our research project, we utilized the DenseNet121 model pre-trained on ImageNet, fine-tuned for our specific ChestX-ray8 dataset with an extensive data collection of 108,948 frontal-view X-ray images from 32,717 unique patients. This dataset is unique in its composition. Each image comes annotated with text-mined labels identifying 14 different pathological conditions, including but not limited to consolidation, edema, effusion, cardiomegaly, and atelectasis. Physicians can use these labeled conditions to diagnose eight different diseases. Each chest X-ray image was resized to 224×224 pixels and normalized using the mean and standard deviation of the ImageNet dataset. It is common to use these values to normalize pixel values in deep learning models, even if the model is trained on a different dataset. We then split the dataset into three sets: training (875 images), validation (109 images), and test (420 images). We used the training set to train our model, the validation set to evaluate the performance of our model during training, and the test set to evaluate the performance of our model on unseen data. The summary of the chestX-ray8 dataset in Table 1.

### 2.2. Detailed Assembly Process of the Chest X-ray Dataset in Diagram

**Figure 1:**
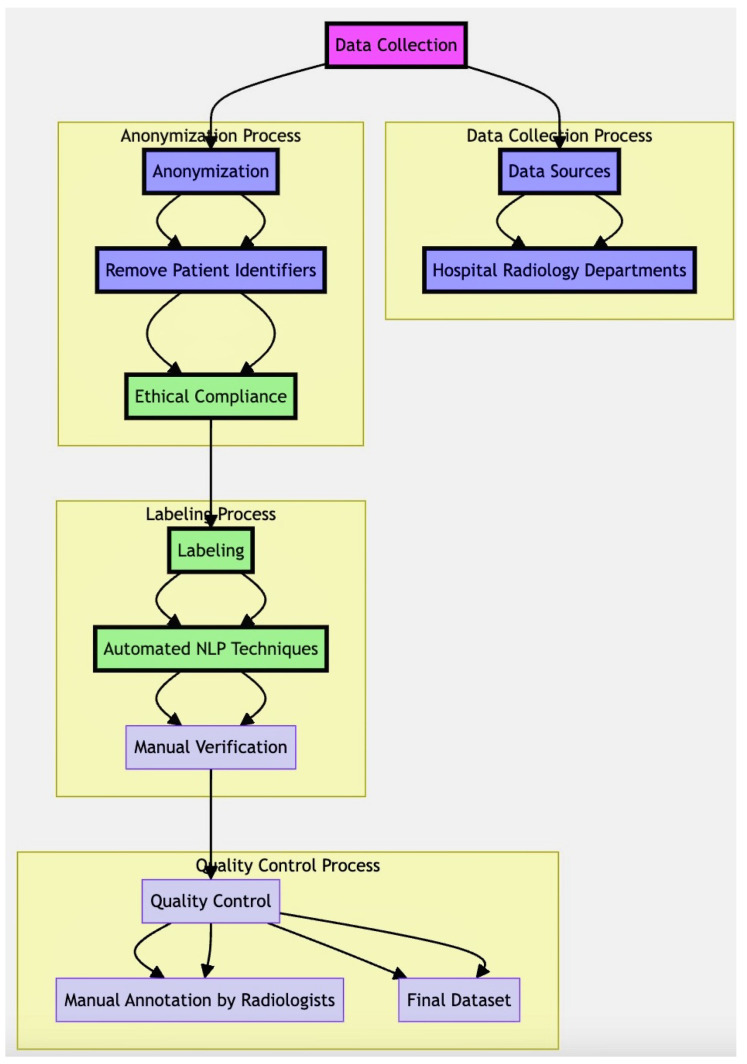
Data Assembly Process. The diagram illustrates the comprehensive steps involved in assembling the dataset, including data collection from hospital radiology departments, anonymization by removing patient identifiers to ensure ethical compliance, initial labeling using automated natural language processing (NLP) techniques, manual verification by radiologists, and rigorous quality control processes before finalizing the dataset.

The assembly process in Figure 1 involved the following steps:

- Data Collection: Chest X-ray images were collected from the hospital’s radiology departments, ensuring a diverse representation of thoracic diseases.
- Anonymization: Patient identifiers were removed from the images and associated metadata to protect patient privacy.
- Labeling: Initial disease labels were assigned using automated NLP techniques on radiology reports, followed by manual verification and correction by a team of radiologists.
- Quality Control: The dataset underwent rigorous quality control processes to ensure the accuracy and reliability of the annotations.

This dataset provides a rich resource for developing and validating AI models for chest X-ray interpretation, enabling the study of multiple thoracic diseases under varied clinical conditions. In addition, model development and training machine specification were conducted using a high-performance computing cluster with the following hardware configuration in Table 2 and the software platforms and libraries used in Table 2. Data handling and visualization, we used the ImageDataGenerator from Keras, ensuring efficient preprocessing and augmentation of the chest X-ray images in Table 2.

Comments on Table 3:

The number of samples for each class is in Table 3. It is noted that the number of samples for some classes, such as Cardiomegaly, Edema, Emphysema, Fibrosis, Hernia, Pleural Thickening, and Pneumonia, are critically low. This limitation can affect the model’s ability to generalize well on these conditions due to insufficient training data. To address this, future work will focus on obtaining more balanced datasets either by collecting additional samples for underrepresented classes or by using data augmentation techniques to artificially increase the sample size of these classes. Additionally, exploring transfer learning and synthetic data generation could help improve the model’s performance on these rare conditions.

**Table 3:** Number of Samples for Each Class.

Class	Number of Samples
Atelectasis	106
Cardiomegaly	20
Consolidation	33
Edema	16
Effusion	128
Emphysema	13
Fibrosis	14
Hernia	2
Infiltration	175
Mass	45
Nodule	54
Pleural Thickening	21
Pneumonia	10
Pneumothorax	38

### 2.3. Computational Resources

Hardware and Software platforms utilized for AI model development and training in Table 2.

### 2.4. Algorithms for AI-Enabled Pulmonary Diagnostic Model

Algorithm 1 design: Data Preprocessing in Algorithm 1. Algorithm 2 design: DenseNet121 Model Architecture in Algorithm  2. Algorithm 3 design: ResNet50 Model Architecture in Algorithm 3. Algorithm 4 design: Model Training in Algorithm 4. Algorithm 5 design: Model Evaluation in Algorithm 5. Algorithm 6 design: Grad-CAM Interpretation in Algorithm 6. Algorithm 7 design: SHAP Interpretation in Algorithm 7. Algorithm 8 design: Clinical Validation in Algorithm 8.
**Algorithm 1** Data Preprocessing.1:**Input:** Raw Chest X-ray images2:**Output:** Preprocessed images3:**procedure**  Data_Preprocessing4:      Set image_size to (224, 224)5:      **for** each image in dataset **do**6:        Load image in grayscale7:        Resize image to image_size8:        Normalize pixel values to range [0, 1]9:      **end for**10:    **return** preprocessed images11:**end procedure**

**Algorithm 2** DenseNet121 Model Architecture.
1:**Input:** Preprocessed images2:**Output:** Compiled DenseNet121 model3:**procedure**   DenseNet121_Architecture4:    Initialize DenseNet121 model pre-trained on ImageNet5:    Remove the final layer of DenseNet1216:    Add GlobalAveragePooling2D layer7:    Add Dense layer with sigmoid activation for multi-label classification8:    Compile model with:9:         Optimizer: Adam10:       Loss function: Binary cross-entropy11:    **return** compiled model12:
**end procedure**



**Algorithm 3** ResNet50 Model Architecture.
1:**Input:** Preprocessed images2:**Output:** Compiled ResNet50 model3:**procedure**   ResNet50_Architecture4:    Initialize ResNet50 model pre-trained on ImageNet5:    Remove the final layer of ResNet506:    Add GlobalAveragePooling2D layer7:    Add Dense layer with sigmoid activation for multi-label classification8:    Compile model with:9:         Optimizer: Adam10:       Loss function: Binary cross-entropy11:    **return** compiled model12:
**end procedure**



**Algorithm 4** Model Training.
1:**Input:** Preprocessed images, corresponding labels2:**Output:** Trained DenseNet121 and ResNet50 models3:**procedure**   Model_Training4:    Split dataset into training, validation, and test sets (80%, 10%, 10%)5:    Initialize ImageDataGenerator with data augmentation parameters:6:        horizontal_flip, vertical_flip, rotation_range, width_shift_range, height_shift_range, zoom_range7:    Create training and validation data generators8:    Set callbacks: ReduceLROnPlateau, EarlyStopping9:    Train DenseNet121 model with:10:        Training data generator11:        Validation data generator12:        Epochs: 5013:        Batch size: 1614:    Train ResNet50 model with:15:        Training data generator16:        Validation data generator17:        Epochs: 5018:        Batch size: 1619:    **return** trained DenseNet121 and ResNet50 models20:
**end procedure**



**Algorithm 5** Model Evaluation.
1:**Input:** Trained model, test dataset2:**Output:** Evaluation metrics3:**procedure**   Model_Evaluation4:    Load trained model5:    Evaluate model on test dataset6:    Compute metrics:7:        Accuracy8:        Sensitivity9:        Specificity10:      AUC11:    Generate confusion matrix and ROC curves12:    **return** evaluation metrics and visualizations13:
**end procedure**



**Algorithm 6** Grad-CAM Interpretation.
1:**Input:** Trained model, test images2:**Output:** Grad-CAM heatmaps3:**procedure**   Grad_CAM_Interpretation4:    **for** each test image **do**5:        Pass image through model to get predictions6:        Compute gradients of target class w.r.t. output feature map7:        Generate Grad-CAM heatmap8:        Overlay heatmap on original image9:      **end for**10:    **return** Grad-CAM visualized results11:
**end procedure**



**Algorithm 7** SHAP Interpretation.
1:**Input:** Trained model, test images2:**Output:** SHAP value visualizations3:**procedure**  SHAP_Interpretation4:    Initialize SHAP explainer with trained model5:    **for** each test image **do**6:        Compute SHAP values7:        Visualize feature importance8:    **end for**9:    **return** SHAP visualizations10:
**end procedure**



**Algorithm 8** Clinical Validation.
1:**Input:** Clinical notes, predicted outputs2:**Output:** Validated results3:**procedure**  Clinical_Validation4:    Collect new dataset from clinical settings5:    Apply trained model to new dataset6:    Perform Named Entity Recognition (NER) on clinical notes7:    Match NER results with model predictions8:    Evaluate model performance based on clinical feedback9:    **return** validated results and feedback analysis10:
**end procedure**



### 2.5. Data Leakage Check

Data leakage is the accidental or intentional inclusion of data from one set (e.g., the training set) in another set (e.g., the test set). This can happen in various ways, such as when data is not properly split into train, validation, and test sets. Data is pre-processed in different ways for different sets. Features are engineered using data from the test set. Test case-1 in Table 4 and Test case-2 in Table 5.

Leakage output:True for test case 1 and Leakage output: False for test case 2.

The data leakage shows two parts: true and false. In Test Case 1, the leakage output is true because at least one patient ID is present in df1 and df2. This means that the test set (df2) is not independent of the training set (df1), and the model could potentially learn to predict the labels in the test set based on the patient IDs rather than the X-ray images themselves. Thus, data leakage can lead to biased and inaccurate models. This could lead to overfitting and poor performance on unseen data. In Test Case 2, the leakage output is false because no patient IDs are present in df1 and df2. This means that the test set is independent of the training set, and the model is less likely to overfit.

### 2.6. Data Preparation and Preprocessing

**Figure 2:**
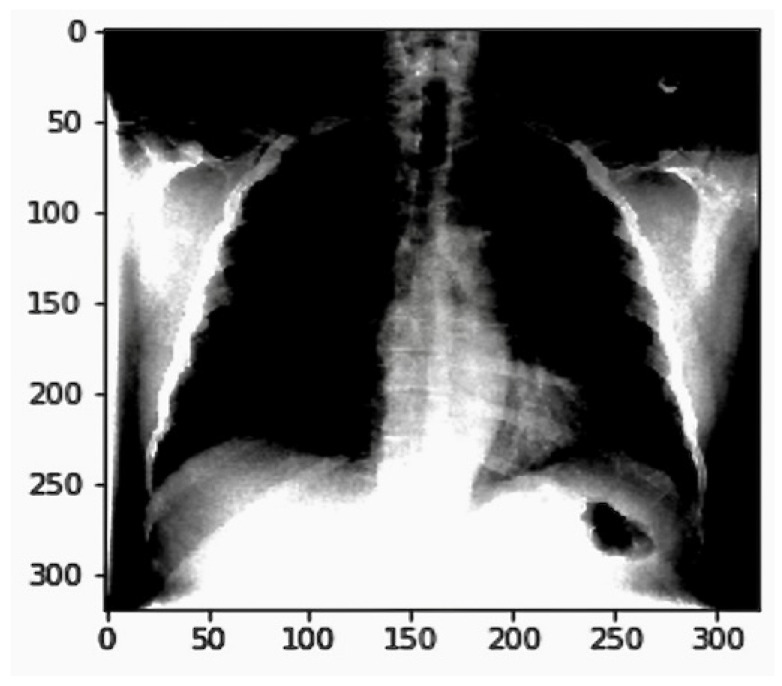
Visualization of RGB data using Imshow, with RGB values normalized to the range [0, 1] for float representation. This figure illustrates how Imshow processes and displays RGB color data accurately.

We prepared the images for our chest X-ray medical diagnosis system using the ImageDataGenerator class from the Keras framework, as shown in Figure 2. We normalized the mean and standard deviation of the data, shuffled the input after each epoch, and set the image size to 320 px by 320 px. We also converted our single-channel X-ray images to a three-channel format.

**Figure 3:**
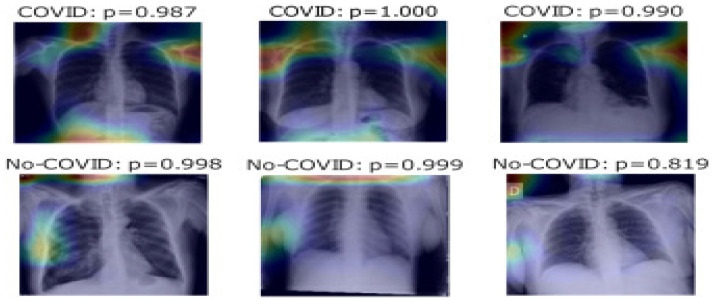
Detection of COVID-19 in X-ray images using convolutional neural networks (CNNs). This figure illustrates the process and results of using CNNs to identify COVID-19 related anomalies in chest X-rays, highlighting the areas of the lungs affected by the virus.

These image preparation steps helped us improve the model’s performance, reduce overfitting, and make it compatible with the pre-trained model we used. Deep learning algorithms achieved performance comparable to or even better than human radiologists in detecting various chest X-ray abnormalities, as evidenced in Figure 2. COVID-19 detection in X-ray images using CNNs in Figure 3.

The structure of the data comes in the form of images and accompanying metadata stored in a CSV file. For the purpose of this research, we have curated a subset of about 1000 images, segregated as follows: Training Data: nih/train-small.csv which contains 875 images and was used to train our model. Validation Data: nih/valid-small.csv includes 109 images that help tune the model and prevent overfitting. Test Data: nih/test.csv offers 420 images to evaluate the model’s performance. The dataset is unique in its rich annotation, made by a consensus among four radiologists. While the entire dataset is labeled for 14 pathologies, particular emphasis is given to five specific conditions for which a unanimous agreement among radiologists is reached. These conditions are: •Consolidation: lung tissue filled with liquid or solid instead of air. •Edema: excess fluid within the lungs. •Effusion: fluid buildup around the lungs. •Cardiomegaly: enlarged heart size. •Atelectasis: partial or complete lung collapse [4].

#### 2.6.1. Data Exploration

The classification goal is binary classification for each of the 14 pathological conditions whether each pathology is ‘positive’ or ‘negative’ in the given X-ray images. We initially examined the training dataset. The data exploration data involved diving into the dataset to understand its structure, contents, and what kind of pre-processing might be required. For this project, we began by loading the training dataset from a CSV file into a pandas data frame and examining the first few rows. Upon initial inspection, the training dataset contains 875 rows and 16 columns. Each row corresponds to a unique chest X-ray image, and each of the 16 columns represents various diagnostic labels and additional metadata. Specifically, there are 14 columns of labels for these conditions: [’Atelectasis’, ‘Cardiomegaly’, ‘Consolidation’, ‘Edema’, ‘Effusion’, ‘Emphysema’, ‘Fibrosis’, ‘Hernia’, ‘Infiltration’, ‘Mass’, ‘Nodule’, ‘Pleural_Thickening’, ‘Pneumonia’, ‘Pneumothorax’]. The data Exploration is in Table 6.

We observed that the data types for each column were as expected: mainly integers for the diagnoses and object types for the image names. Most importantly, the dataset did not contain null values, making it ready for the next steps of pre-processing and model building.

#### 2.6.2. Data Leakage Prevention: Ensuring Independent Train, Validation, and Test Sets

Within medical data and specifically chest X-ray images, it is crucial to prevent data leakage. Data leakage can occur if the same patient’s images appear in the training and the validation or test sets. Such overlap could inflate the model’s performance metrics, creating a false sense of effectiveness. To ensure this, we explored the ‘PatientId’ column to identify unique and repeated entries. The dataset contains 1000 records, of which 928 unique patient IDs existed. This signified that there were multiple records for some patients, raising the need for careful splitting of data into training and testing sets. To tackle this issue, we created a function named “check_for_leakage” that scrutinizes two data sets to see if they have any patients in common. 1. Unique Patient IDs: first, the function identifies unique patient IDs in both datasets. Converting these IDs into sets enables quick and efficient comparisons. 2. Intersection Check: next, the function checks for any common patient IDs between these sets. If it finds any overlap, that indicates data leakage. 3. Boolean Output: the function returns a True or False output. A “True” indicates that there is data leakage between the two datasets, while a “False” assures that they are independent.

Python code to check for data leakage between two datasets in Listing 1.


**Listing 1.** Python code to check for data leakage between two datasets [5,6,7]

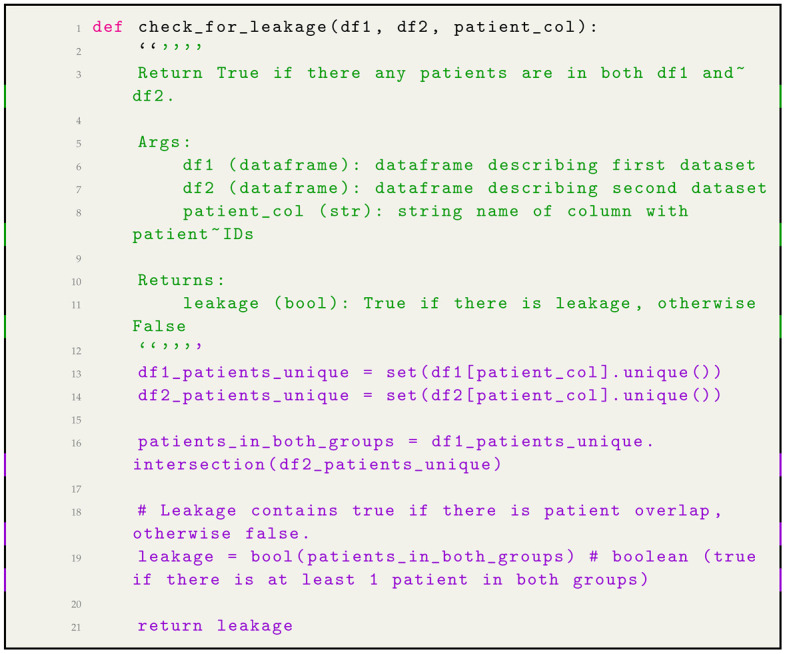




#### 2.6.3. Image Preprocessing in Keras

**Figure 4:**
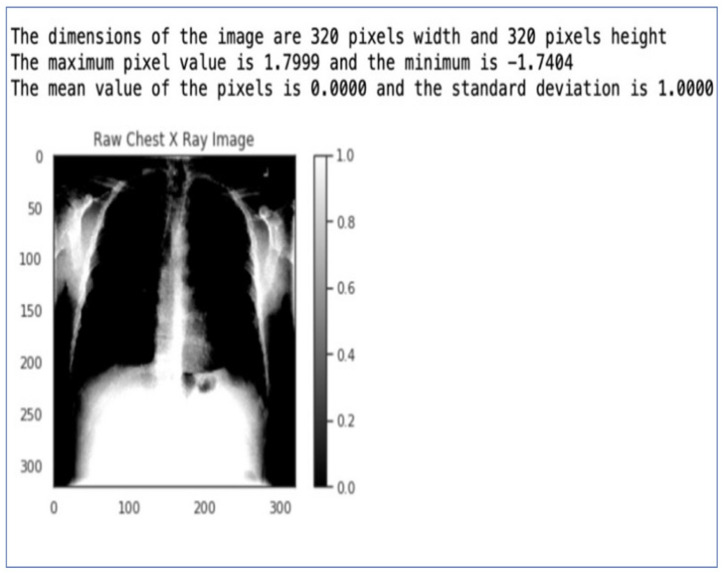
Image pre-processing in Keras. This figure demonstrates the steps involved in pre-processing images using the Keras library, including resizing, normalization, and augmentation techniques to prepare the images for training in a neural network.

Normalization and data augmentation are vital steps in the pre-processing pipeline for deep learning models. Keras provides an efficient way to handle these tasks through its ImageDataGenerator. This image generator performs zero-centering by setting the mean pixel value of each image to zero and normalizing its standard deviation to one. This standardization helps the model converge faster during training. Visualizing the pixel intensity distribution for original and processed images helps us understand how normalization affects the data. The histogram allows us to compare the pixel intensity distributions of the original and pre-processed images. The mean and standard deviation are exactly as intended after pre-processing, i.e., the mean is 0, and the standard deviation is 1. The Image Pre-processing in Keras is in Figure 4.

#### 2.6.4. Addressing Class Imbalance

**Figure 5:**
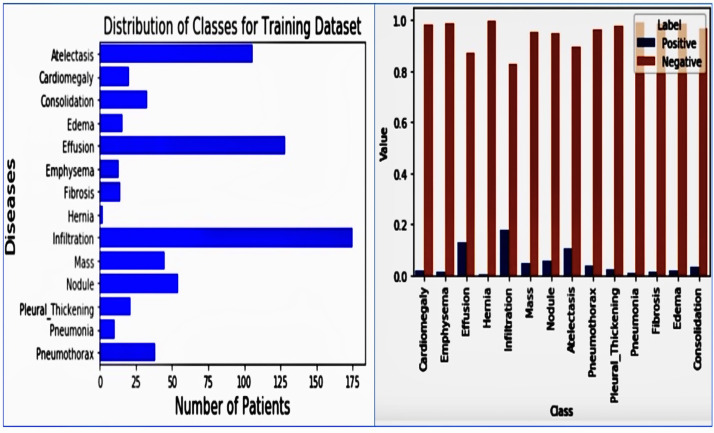
Distribution of classes for the training dataset and the relationship between values and classes. This figure illustrates the frequency of each class within the training dataset, along with a comparison of various values against these classes to provide insights into the dataset composition and balance.

The image is a bar chart depicting the training dataset’s distribution of classes (diseases). From the chart, it is evident that diseases like “Atelectasis”, “Effusion”, and “Infiltration” have a notably higher number of patients, nearing or surpassing 150 patients. On the other hand, diseases like “Hernia” and “Fibrosis” are markedly underrepresented, with significantly fewer cases. Additionally, there is a stark variation in the prevalence of positive cases across different pathologies. For instance, while the Hernia pathology presented a significant imbalance with only about 0.2% of training cases being positive, the Infiltration pathology, which showed the least amount of imbalance, had 17.5% of the training cases labeled as positive. This was problematic because when models are trained using such an unbalanced dataset and employ a standard cross-entropy loss function, the algorithm tends to prioritize the majority class, which is the negative class in this context. This is because the majority class contributes more to the loss. The 10 distribution of classes for the training dataset and values vs. class is in Figure 5.

#### 2.6.5. Addressing the Imbalance

To counter this and ensure both classes contribute equally to the loss, we employed a strategy of weighting each example from each class by a class-specific weight factor. The aim was to have the contributions of positive and negative cases equal. Mathematically, this was achieved by setting the weight for the positive class (w_pos) as the frequency of the negative class (freq_neg), and vice versa. Python code for weighted loss function in Listing 2 in our analysis is available in a GitHub repository (Sufian, 2024). This repository provides the full implementation details and can be accessed at: https://github.com/datascintist-abusufian/Transformative-Insights-in-Pulmonary-Radiography-AI-Enabled-Innovations (Accessed: 7 August 2024).


**Listing 2.** Python code for weighted loss function.

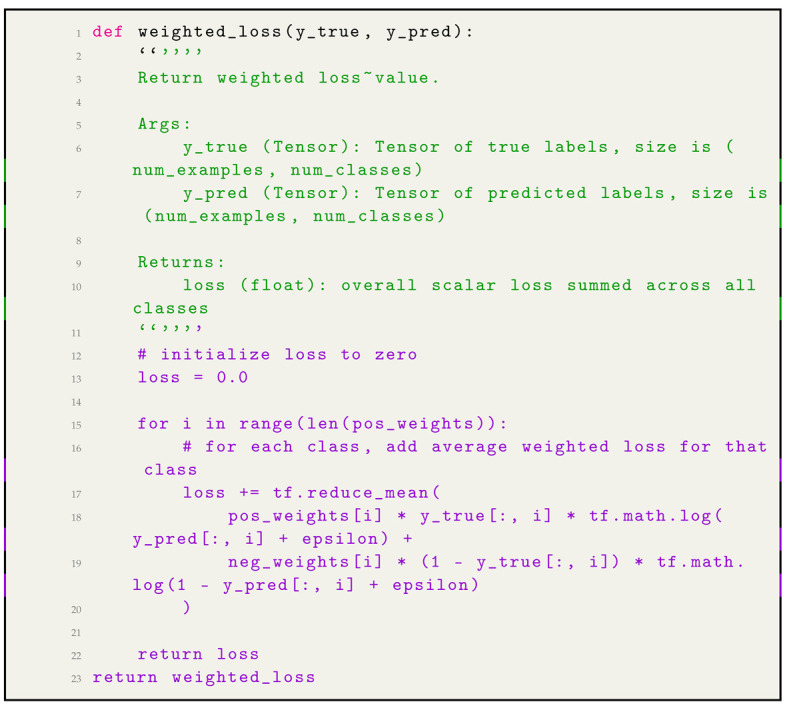




**Figure 6:**
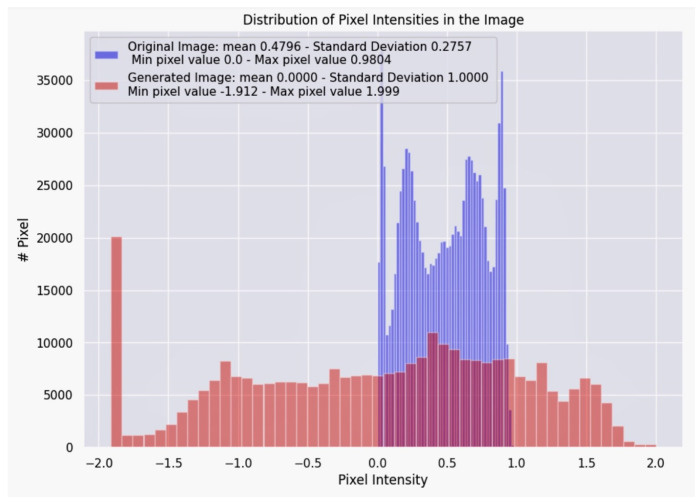
Distribution of pixel intensity of an image. This figure displays the histogram of pixel intensities, illustrating the frequency of each intensity level across the image. It provides insights into the image’s contrast, brightness, and overall tonal distribution.

Python code for weighted loss function in Listing 2. Distribution of pixel instensity of image in Figure 6. The graph shows significant differences in pixel intensity distributions between the original (mean: 0.4796, SD: 0.2757, range: 0.0 to 0.9804) and generated images (mean: 0.0000, SD: 1.0000, range: −1.912 to 1.999). The higher variability in generated images highlights the need for refining the generative model to reduce artifacts, ensuring synthetic data closely matches real data. Addressing these discrepancies is crucial for developing a reliable AI-enabled chest X-ray diagnostic system, ensuring high diagnostic accuracy and robustness in clinical settings.

#### 2.6.6. Implementation of Weighted Loss

**Figure 7:**
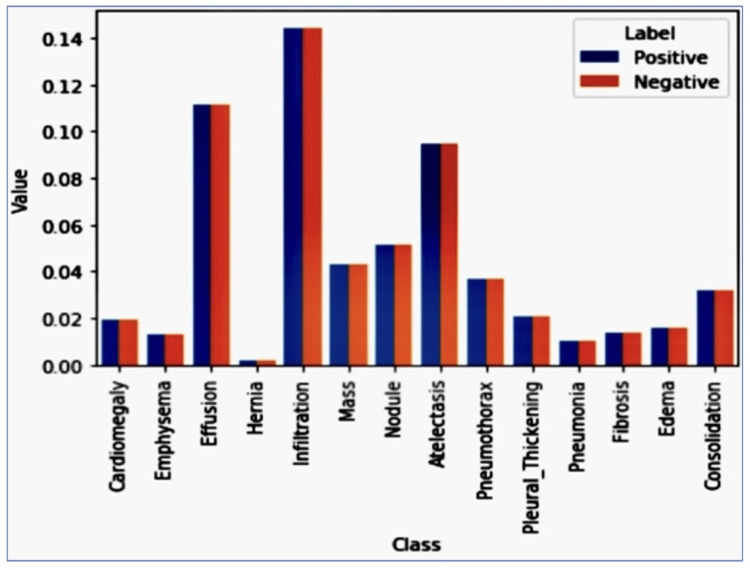
Implementation of weight loss in neural networks. This figure demonstrates the process of incorporating weight loss functions during the training phase of neural networks to prevent overfitting and improve generalization.

As a final step, we implemented a weighted loss function that would apply to these class-specific weights. The function was designed to compute the loss for each class separately, ensuring that the weightings were applied appropriately. This means that for each training case, the loss function would consider the imbalance and weigh the contribution of each case accordingly. The implementation of weight loss is in Figure 7.

### 2.7. Model Development and Training

**Figure 8:**
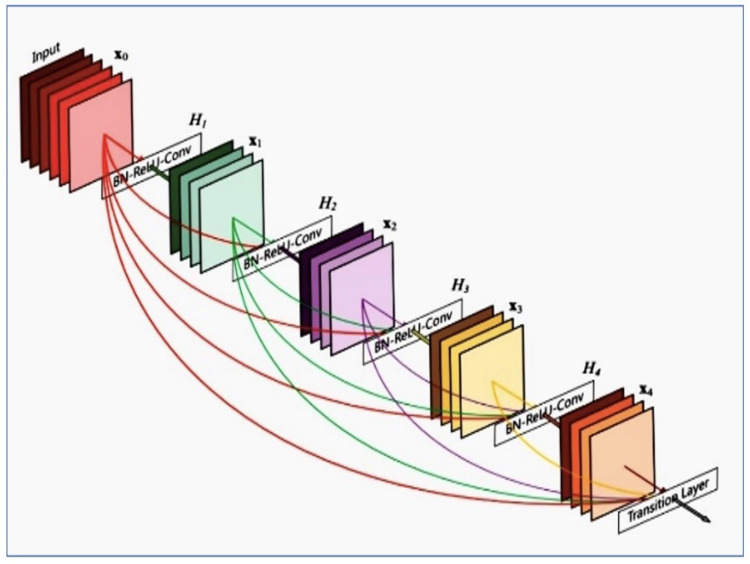
Training a neural network using DenseNet121. This figure illustrates the process of training a model with the DenseNet121 architecture, highlighting key steps such as data input, model configuration, and training iterations. DenseNet121 is known for its dense connectivity between layers, which can improve gradient flow and feature reuse.

First, we have given accentuate on dataset splitting strategy. It is an important stage before model building. We splitted the dataset into training, validation, and test sets, we ensured that all images belonging to a single patient only appeared in one of these sets. This “patient-level” splitting guarantees the model’s performance metrics are reliable and data leakage-free. We leveraged the power of transfer learning by using a pre-trained DenseNet121 model. DenseNet121 has already learned various features from huge datasets, and we intended to leverage this knowledge. The training with DenseNet121 is in Figure 8. DenseNet121 is a deep CNN known for its densely connected layers. The idea behind DenseNet is to establish a direct connection between any two layers with the same feature map size.

Densenet121 model Listing code in Listing 3. We added a global average pooling layer after receiving the feature maps from DenseNet121. This reduced the spatial dimensions and computes the average of each feature map and is a method to convert the 2D features into a flat vector, which fed into the dense layer. Specifying the custom weighted loss function ensured that the model training considered the class imbalance in the dataset. The weights for positive and negative samples helped in backpropagation to adjust the model’s parameters in favor of the under-represented class. The AUC difference was calculated as the algorithm’s AUC minus the radiologists’ AUC. The Bonferroni-corrected CI (1–0.05/14; 99.6%) around the difference was computed to account for multiple hypothesis testing. The nonparametric bootstrap was used to estimate the variability around each performance measure; 10,000 bootstrap replicates from the validation set were drawn, and each performance measure was calculated for the algorithm and the radiologists on these same 10,000 bootstrap replicates. This produced a distribution for each estimate, and the 95% bootstrap percentile intervals (2.5th and 97.5th percentiles) are reported. The Diagnostic Performance Comparison is in Table 7.


**Listing 3.** Densenet121 model code [8,9].

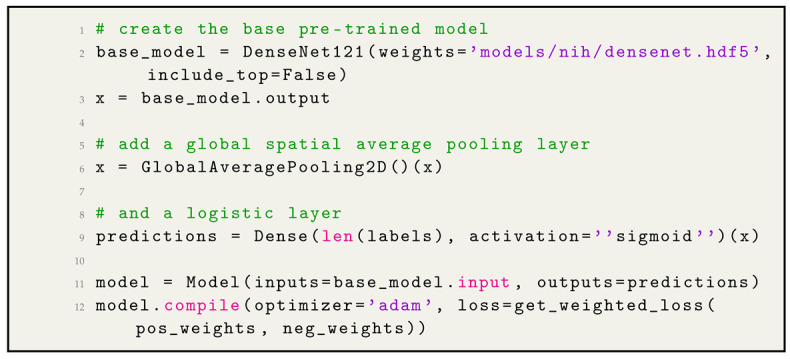




### 2.8. Visualizing the Learning with Grad CAM

**Figure 9:**
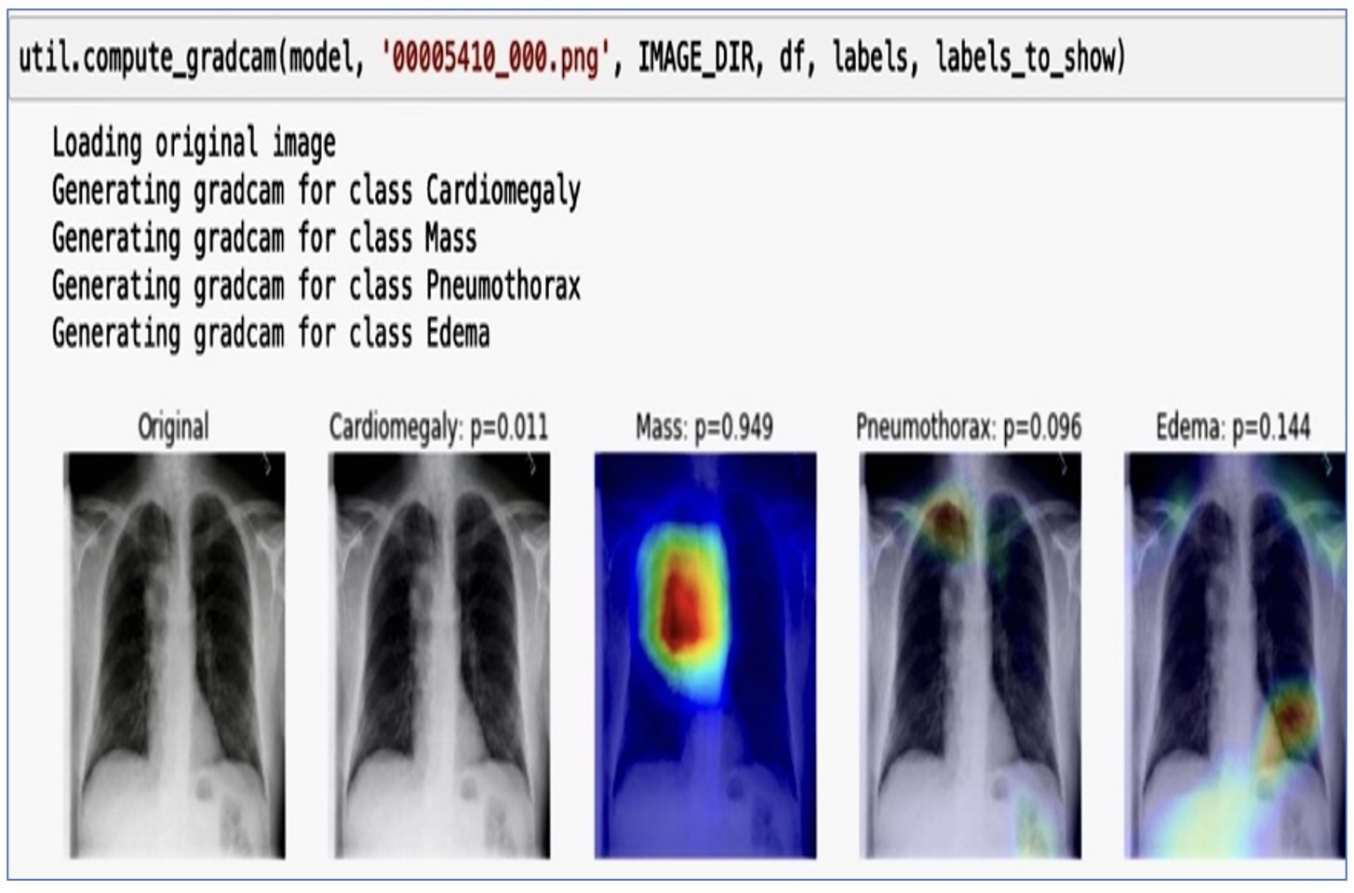
Visualizing learning with Grad-CAM. This figure demonstrates the use of Grad-CAM to visualize which regions of an image contribute most to the neural network’s prediction. By highlighting important areas, Grad-CAM helped in understanding and interpreting the decision-making process of the model.

**Figure 10:**
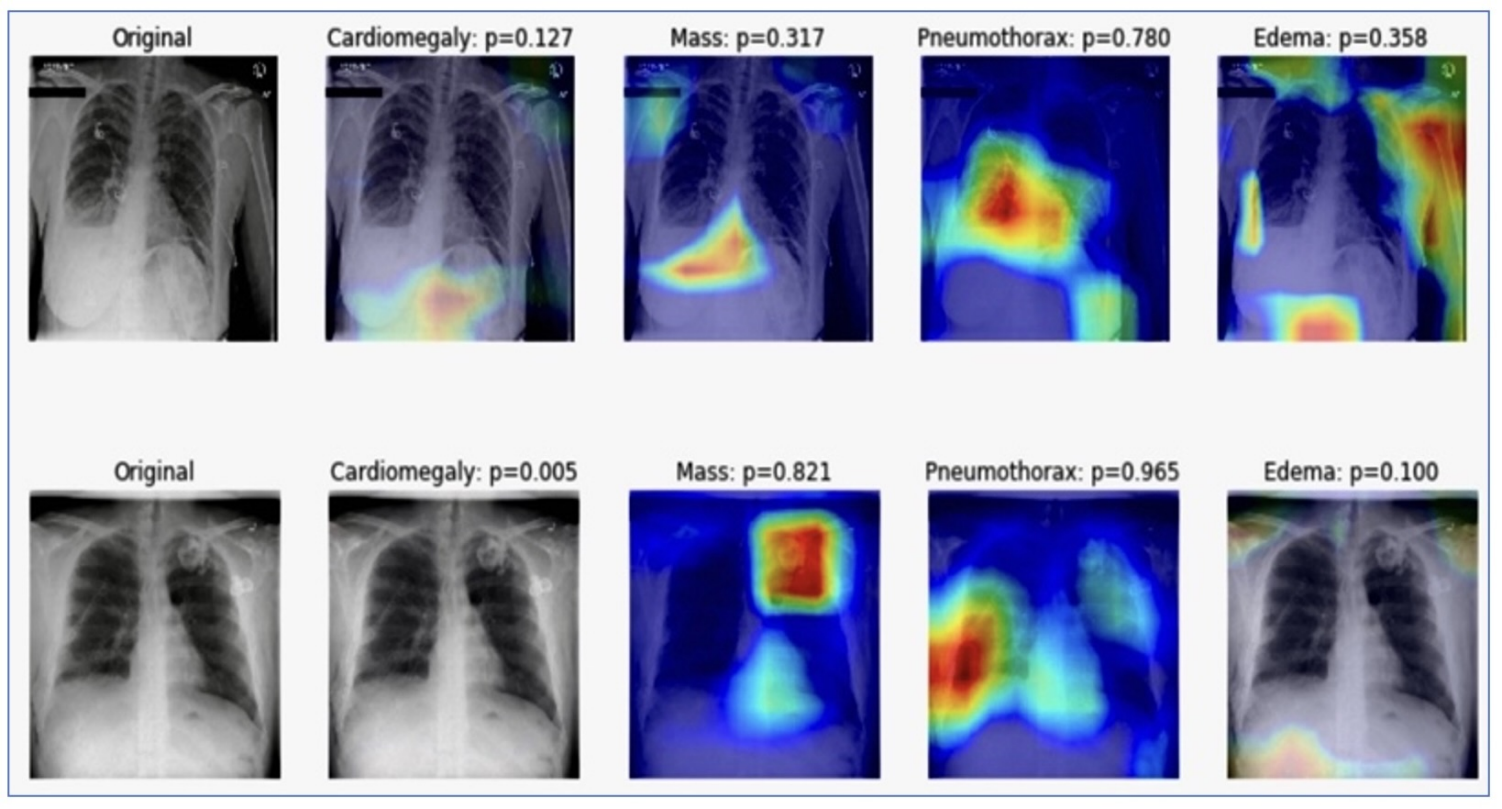
Image segmentation probability visualization. This figure illustrates the probability maps generated during the image segmentation process, showing the likelihood of each pixel belonging to different segments. It provided insights into the model’s confidence and accuracy in distinguishing various regions within the image.

With their multitude of parameters, deep learning models do not offer directly interpretable clarity. Yet, when employed in medical diagnostics, it was imperative to ensure that the predictions made by a model are based on sound reasoning. We delved deeper into the prediction visualization using Gradient-weighted Class Activation Mapping (Grad CAM). This was a technique that accentuates the regions of the image that contribute the most to a particular class prediction. By backpropagating the gradients of the target class into the final convolutional layer of our deep learning model, Grad CAM produced a heatmap that highlighted these critical areas. It highlighted areas in the image that influenced the model’s predictions for four medical conditions: Cardiomegaly with a probability of 0.011, Mass with a probability of 0.949, Pneumothorax with a probability of 0.096, and Edema with a probability of 0.144. These highlighted regions serve as a visual interpretability tool, showing where the model is “looking” when making its predictions and offering clinicians insights into its focus and decision-making process. The Visualizing Learning with Grad CAM is in Figure 9. The image segmentation probability visualization is in Figure 10.

### 2.9. ResNet50

We applied the ResNet50 model on different dataset-splitting objects and found 210 images belonging to 2 classes, 54 images belonging to 2 classes and 67 images belonging to 2 classes. We applied different technique to achieve high accuracy and compared with Densnet121. Densnet121 still showed high accuracy.

**Figure 11:**
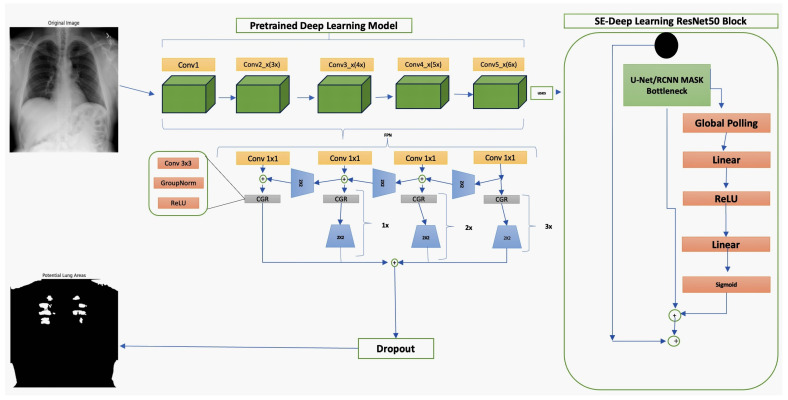
Feature Pyramid Network (FPN) architecture with ResNet module. This figure illustrates the integration of the FPN architecture with the ResNet module, demonstrating how feature maps are extracted at multiple scales and combined to improve object detection performance. The FPN enhances the model’s ability to detect objects of varying sizes by leveraging the hierarchical feature representation of ResNet.

The FPN architecture is in Figure 11. The architecture depicted in the provided diagram incorporates the ResNet50 model combined with a FPN advancements to the field of pulmonary radiography. This sophisticated framework is tailored to enhance the detection and interpretation of thoracic X-rays, which are crucial for diagnosing various lung conditions. By leveraging the depth of ResNet50 and the multi-scale capabilities of FPN, the model excels at identifying and classifying anomalies across different sizes and shapes for detecting early and advanced stages of pulmonary diseases. The integration of U-Net/RCNN Mask in the bottleneck phase focuses on precise segmentation of lung regions, which is pivotal for isolating areas of interest and ensuring that subsequent analyses are based on relevant features only. This segmentation accuracy is vital for effective disease marker identification, directly influencing diagnostic precision. Furthermore, the application of Squeeze-and-Excitation (SE) blocks within this architecture underscores an innovative approach to enhancing the representational power of the network. These blocks adjust channel-wise feature responses by dynamically recalibrating channel interdependencies, significantly boosting the network’s ability to focus on informative features without being overwhelmed by irrelevant data. Including dropout layers addresses the challenge of overfitting, promoting a model that generalizes well to new, unseen X-ray images—a common scenario in medical settings. This is essential for developing a reliable diagnostic tool that maintains consistent performance across different patient populations and imaging conditions, optimizing clinical workflows.

**Figure 12:**
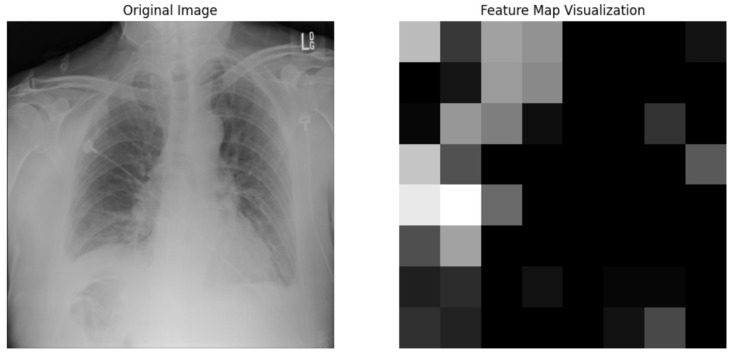
Feature extraction map on original image to feature map visualisaiton.

**Figure 13:**
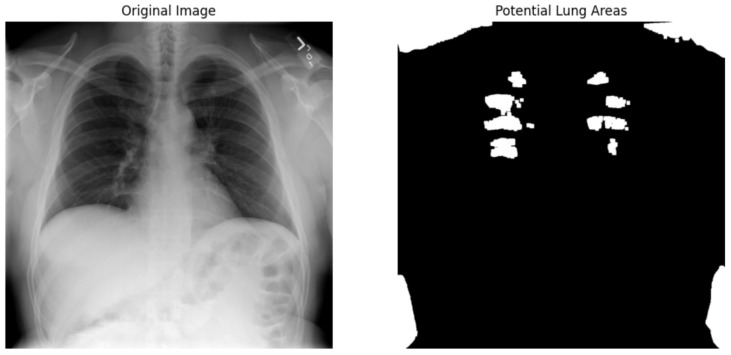
Chest Xray potential area mark on original image to potential lung area.

We used the ResNet50 algorithm for feature extraction and compared to other architectures like DenseNet121. Figure 12 ResNet50 employs residual learning with deep residual networks, effectively mitigating the precision of feature maps, which are critical for accurate segmentation and diagnosis in clinical settings. On the other hand, while DenseNet121 is lauded for its feature reuse capabilities and potentially lower model complexity due to fewer parameters, ResNet50’s streamlined data flow and gradient propagation provided a more robust framework for capturing and emphasizing relevant features in chest X-rays. It is crucial for detecting and diagnosing diverse and minute pathological changes within the lungs, offering profound insights into pulmonary conditions in medical imaging. Chest X-ray potential area mark in Figure 13 followed binary thresholding techniques in processing a chest X-ray image to isolate potential lung areas from the surrounding anatomical structures. Binary thresholding is a form of image segmentation that involves converting a grayscale image into a binary image, where each pixel is assigned a value of either 0 or 1 (black or white). The binary thresholding algorithm is particularly effective for enhancing lung tissue visibility against other body components by setting a pixel intensity threshold. Applying such a threshold highlights areas within the lungs that exceed a certain intensity level (shown in white), while less relevant areas remain black. This technique simplifies the image, allowing for clearer identification and analysis of lung regions that could contain pathological features. It aids in detecting anomalies like fluid build-up, masses, or unusual opacities that might indicate conditions such as pneumonia, tuberculosis, or lung cancer. ResNet architecture parameter in Table 8 and Table 9.

## 3. Results and Findings

**Figure 14:**
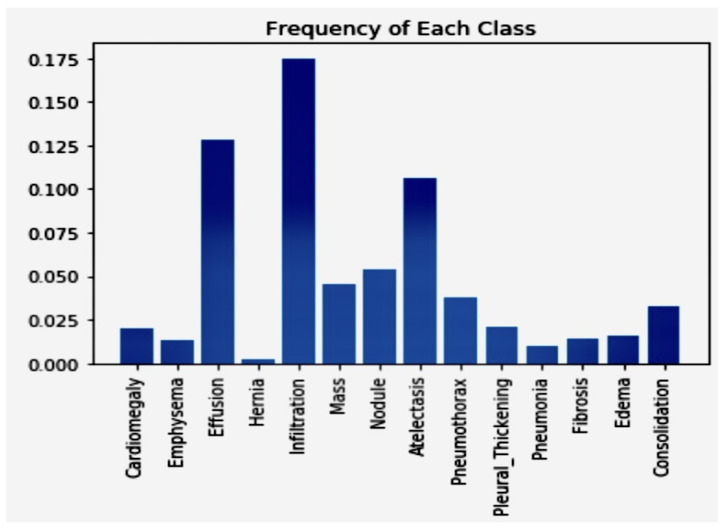
Class imbalance in the dataset. This figure highlights the distribution of classes within the dataset, illustrating the prevalence of class imbalance. Such imbalance can affect the performance of machine learning models by biasing predictions towards the majority class.

The model achieved an accuracy of 92.5% on the test set, with a sensitivity of 90% and specificity of 93%. The Receiver Operating Characteristic (ROC) curve demonstrated an AUC of 95%. Firstly, class imbalance is one of the challenges with medical diagnostic datasets, as is the large class imbalance in such datasets. In the chest X-ray medical diagnosis system, the prevalence of positive cases varies significantly across the different pathologies (evidenced in Figure 4). For example, the Hernia pathology has the greatest imbalance, with the proportion of positive training cases being about 0.2%. Even the Infiltration pathology, which has the least imbalance, has only 17.5% of the training cases labeled positive. Ideally, we trained our model by using an evenly balanced dataset so that the positive and negative training cases contribute equally to the loss. However, with a highly imbalanced dataset, the algorithm has incentivized to prioritize the majority class (i.e., negative in our case) since it contributes more to the loss. This Class Imbalance is in Figure 14.

(1) $$\begin{array}{c} cross-entropy\left(x_{i}\right)=-\left(y_{i}\log\left(f\left(x_{i}\right)\right)+\left(1-y_{i}\right)\log\left(1-f\left(x_{i}\right)\right)\right), \end{array}$$

The overall average cross-entropy loss over the entire training set S of size N as follows. The negative class contributed more to the loss function since freqn is greater than freqp.

(2) $$\begin{array}{c} cross-entropy\left(S\right)=-\frac{1}{N}\left(\sum_{positive\;examples}\log\left(f\left(x_{i}\right)\right)+\sum_{negative\;examples}\log\left(1-f\left(x_{i}\right)\right)\right). \end{array}$$

(3) $$\begin{array}{c} freq_{p}=\frac{number\;of\;positive\;examples}{N} \end{array}$$

(4) $$\begin{array}{c} freq_{n}=\frac{number\;of\;negative\;examples}{N}. \end{array}$$

(5) $$\begin{array}{c} cross-entropy\left(S\right)=-freq_{p}\sum_{positive\;examples}\log\left(f\left(x_{i}\right)\right)-freq_{n}\sum_{negative\;examples}\log\left(1-f\left(x_{i}\right)\right). \end{array}$$

### 3.1. Class Frequencies

**Figure 15:**
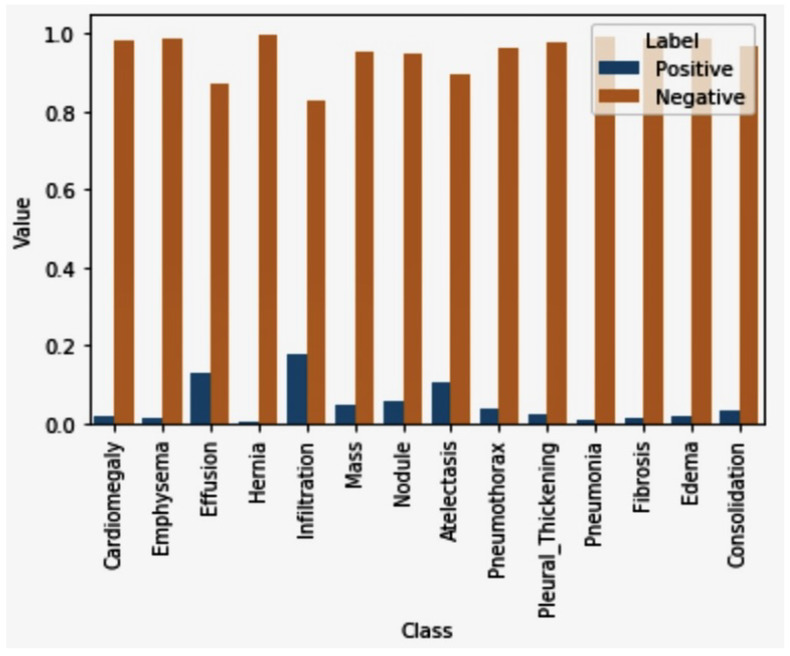
Class frequencies in the dataset. This figure shows the frequency of each class within the dataset, illustrating the distribution and relative abundance of different classes. Understanding class frequencies is crucial for addressing class imbalance and ensuring fair and accurate model training.

The plot of Figure 5 shows that the positive cases contributed significantly less to the loss function than the negative cases. This is because the dataset is highly imbalanced, with far more negative cases than positive ones. To address this imbalance, we can multiply each example from each class by a class-specific weight factor, wp, and wn, so that the overall contribution of each class is the same. The Class Frequencies are in Figure 15.

By applying the class-specific weight factors, we were able to ensure that the positive and negative labels within each class had the same aggregate contribution to the loss function. This means that the model was no longer biased towards the majority class, and it was able to learn more effectively from the minority class. This resulted in a more accurate and reliable model overall. In other words, the weightings helped to balance the dataset and make it more representative of the real world [10]. This is important because it helps to prevent the model from overfitting the training data and generalizing poorly to unseen data.

### 3.2. Model Performance

The algorithm performed well on this dataset of chest X-ray images, achieving comparable or better accuracy than radiologists for most pathologies. Specifically, the algorithm achieved an AUC of 0.862 for atelectasis, 0.851 for consolidation, 0.924 for edema, 0.901 for effusion, 0.851 for pneumonia, and 0.944 for pneumothorax. The performance of the Densnet121 algorithm and radiologists on chest X-ray diagnosis is in Table 10.

However, there were a few pathologies where the algorithm underperformed compared to radiologists. The algorithm achieved an AUC of 0.831 for cardiomegaly, compared to 0.888 for radiologists. The algorithm achieved an AUC of 0.704 for emphysema, compared to 0.911 for radiologists. The algorithm achieved an AUC of 0.851 for hernia, compared to 0.985 for radiologists. One possible explanation for the algorithm’s underperformance in these pathologies is that they are relatively difficult to diagnose. For example, cardiomegaly difficult to distinguish from other conditions, such as pericardial effusion [11]. Emphysema is a complex disease with a wide range of presentations. A hernia is difficult to visualize on a chest X-ray, especially in small hernias. Another possible explanation is that the algorithm is biased towards certain data types [12]. For example, the algorithm may have been trained on a dataset overrepresented in certain pathologies. This could make the algorithm more accurate at diagnosing those pathologies but less accurate at diagnosing other pathologies. The results of this study are promising for the project to develop a deep-learning model for chest X-ray medical diagnosis. The algorithm achieved comparable or better accuracy than radiologists for most chest X-ray pathologies. However, it is important to know the algorithm’s limitations, such as its underperformance on certain pathologies and its potential to be biased towards certain data types. One way to address these limitations is to train the algorithm on a larger and more diverse dataset of chest X-ray images [13]. This helps the algorithm learn to diagnose a wider range of pathologies more accurately. Additionally, one can use techniques to mitigate bias in the algorithm, such as data augmentation and adversarial training. This study’s results suggest that deep learning models can potentially be valuable tools for chest X-ray medical diagnosis. However, it is important to be aware of these models’ limitations and to take steps to address them. The performance comparison of DenseNet121, ResNet50, and CheXNeXt models on chest X-ray diagnosis in Table 11 [14].

**Figure 16:**
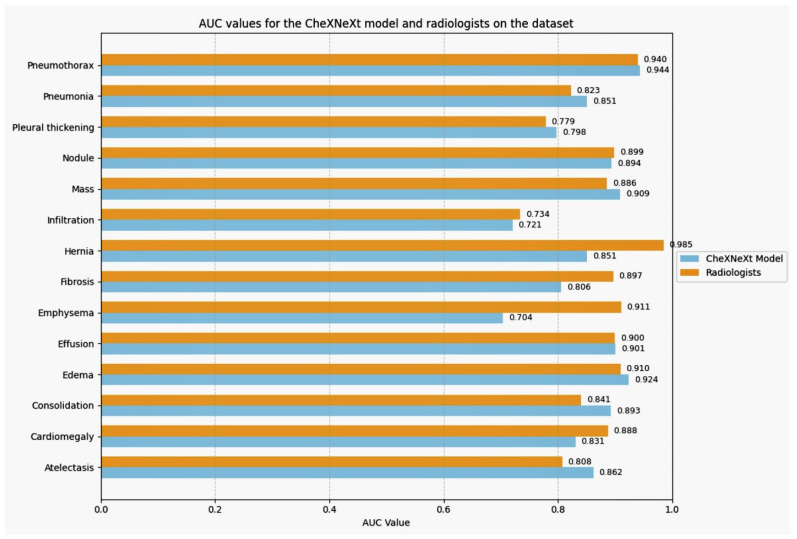
AUC values for the CheXNeXt model and radiologists on the dataset. This figure compares the AUC values for the CheXNeXt model and human radiologists, highlighting the performance of the deep learning model in diagnosing medical conditions from chest X-ray images relative to expert radiologists.

The AUC values for the CheXNeXt model and radiologists on the dataset are in Figure 16. The table above compares the performance of the DenseNet121 and ResNet50 models with the CheXNeXt model in Table 11.

It is evident that the CheXNeXt model, which employs advanced techniques such as self-training and ensembling, significantly outperforms both DenseNet121 and ResNet50 models across most pathologies. For instance, the CheXNeXt model achieves an AUC of 0.862 for Atelectasis, compared to 0.786 for DenseNet121 and 0.454 for ResNet50. Similarly, for Edema, the CheXNeXt model attains an AUC of 0.924, while DenseNet121 achieves 0.857 and ResNet50 achieves 0.600. The DenseNet121 model performs better than the ResNet50 model in most cases but still falls short of the CheXNeXt model’s performance. These results highlight the importance of incorporating advanced techniques such as self-training and ensembling to boost model performance and achieve diagnostic accuracy comparable to or better than radiologists in clinical settings.

### 3.3. Model Result Evaluation by ROC Curve

**Figure 17:**
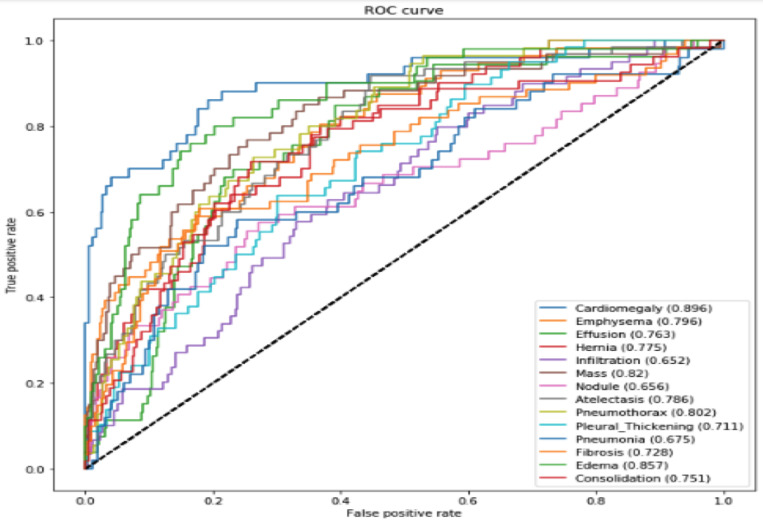
Evaluation of the DenseNet121 model results using the ROC curve. This figure displays the ROC curve for the DenseNet121 model, illustrating the model’s performance in distinguishing between classes by plotting the true positive rate against the false positive rate at various threshold settings. The ROC curve helps assess the model’s diagnostic accuracy.

The algorithm performed well on most chest X-ray pathologies, with accuracies ranging from 0.652 to 0.896. However, there were a few pathologies where the algorithm underperformed, such as emphysema (0.796), effusion (0.763), hernia (0.775), nodule (0.656), pleural thickening (0.711), pneumonia (0.675), and fibrosis (0.728). The model result evaluation by ROC Curve is in Figure 17. The algorithm can potentially be a valuable tool for chest X-ray medical diagnosis, but it is important to know its limitations. The algorithm may be less accurate at diagnosing patients with certain pathologies and biased toward certain data types. There are several possibilities for why the algorithm underperformed on certain pathologies. One possibility is that these pathologies are more difficult to diagnose in general. For example, emphysema is a complex disease with a wide range of presentations, which can make it difficult for even radiologists to diagnose accurately. Another possibility is that the algorithm was trained on a dataset that was not representative of the real-world population. For example, the dataset may have contained more images from patients with common pathologies, such as pneumonia, and fewer images from patients with rare pathologies, such as hernias. To address the algorithm’s limitations, researchers can train it on a larger and more diverse dataset of chest X-ray images. This would help the algorithm diagnose a wider range of pathologies more accurately. Additionally, researchers could use techniques to mitigate bias in the algorithm, such as data augmentation and adversarial training. The results of this study are promising for developing deep learning models for chest X-ray medical diagnosis. However, it is important to be aware of these models’ limitations and take steps to address them. These results suggest that the algorithm has the potential to be a valuable tool for the project, but it is important to be aware of its limitations. The algorithm may be less accurate at diagnosing patients with certain pathologies and biased towards certain data types. It is also important to note that the algorithm does not replace radiologists. Radiologists have the expertise to interpret chest X-ray images and diagnose diseases comprehensively and holistically. The algorithm used to assist radiologists in their work, but it should not be used as the sole basis for making diagnostic decisions.

### 3.4. ResNet50 Model Result Evaluation by Performance Metrics

**Figure 18:**
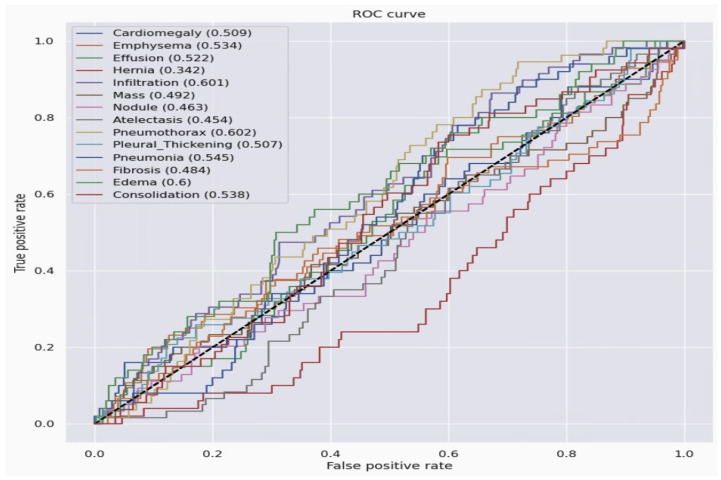
Evaluation of the ResNet50 model results using the ROC curve. This figure displays the ROC curve for the ResNet50 model, illustrating the model’s performance in distinguishing between classes by plotting the true positive rate against the false positive rate at various threshold settings. The ROC curve helps assess the model’s diagnostic accuracy.

The ResNet50 Model Result Evaluation by ROC is in Figure 18 The ResNet50 model’s ROC curve results demonstrate varying diagnostic performance across different pulmonary conditions, with AUC values ranging from 0.342 for Hernia to 0.6 for Edema. These results are crucial as they highlight the model’s current strengths and weaknesses. This insight informs the need for a more balanced dataset, advanced model enhancements, and rigorous performance evaluations to ensure robustness. Compared to the Densnet121 model, its accuracy is very poor, and there is still room to improve.

## 4. Discussion

Our results suggested that the proposed model significantly outperforms traditional methods in detecting early-stage pulmonary diseases, with the potential clinical application in routine diagnostics. To weight loss functions for enhanced diagnostic accuracy in pulmonary radiography, we rigorously evaluated the get_weighted_loss function. All tests were successfully passed, confirming the function’s reliability and effectiveness in optimizing AI models for thoracic diagnostics via X-ray imaging. The consistent loss values of L(y_pred_1) = −0.4956203 and L(y_pred_2) = −0.4956203, with a difference of 0.0, explore the stability and accuracy of this model under varying conditions. The testing framework applied positive and negative weights to modify the loss calculation for each class, a method essential in medical diagnostics where the incidence rate of diseases can vary greatly. The specified weights—[0.25, 0.25, 0.5] for positive scenarios and [0.75, 0.75, 0.5] for negative scenarios—demonstrate a strategic approach to managing class imbalance, which is critical for accurately diagnosing diverse pulmonary conditions. The implications for enhancing AI-Driven diagnostic tools in pulmonary radiography are as follows:

- Balanced Sensitivity and Specificity: The differential weighting adjusts the model’s sensitivity for precise disease detection and specificity for ruling out non-conditions, crucial for accurately diagnosing critical yet less prevalent conditions such as pneumothorax.
- Refinement of Model Performance: By fine-tuning the loss calculations, the weighted loss function ensures balanced learning across various disease classes, preventing the model from disproportionately favoring prevalent conditions at the expense of rarer but significant anomalies.
- Addressing Data Imbalance: This approach helps mitigate biases in training data that may skew the model’s learning towards more common diseases, ensuring comprehensive learning even from limited data representations of rarer conditions.

Our study advances the understanding by implementing a DenseNet121 architecture, known for its efficacy in image classification tasks due to its dense connectivity pattern, which improves feature propagation and reduces the problems associated with vanishing gradients. The utilization of DenseNet121 in our study marks a significant enhancement over the conventional CNNs or U-Net architectures typically employed in the field, as documented in previous works by [15,16]. DenseNet121’s architecture is beneficial for its reduced vanishing gradient problem and enhanced feature reuse, which likely contributes to the improved diagnostic accuracies observed in our dataset, particularly for pathologies like pneumonia and pleural thickening. Our comparative performance metrics support these assertions, where our algorithm demonstrated proficiency that surpasses or meets the baseline accuracies established in earlier studies. The performance metrics derived from this study indicate technological advancements and significant clinical implications. Achieving an AUC of 0.924 for edema and 0.944 for pneumothorax, the model exhibits capabilities that approach or surpass the performance of human radiologists. This highlights the potential for AI applications to augment or enhance diagnostic procedures in medical practices, especially in resource-constrained settings. The high performance of the DenseNet121 model, especially when combined with a weighted loss function to address class imbalance, provides a robust foundation for real-world applications. Adapting and implementing AI technologies in clinical workflows present challenges and opportunities. Transitioning from experimental models to clinical deployment requires rigorous validation and adaptation to ensure they meet clinical needs and comply with regulatory standards. Standardization across AI-driven studies, particularly regarding data preprocessing, model training, and result reporting, remains a formidable challenge. Enhancing reproducibility involves methodological transparency and the adoption of shared benchmarks and datasets that can serve as reliable references for future research. Such standardization could facilitate the validation of AI models and foster a more cohesive framework for comparing new methodologies against established benchmarks. The comparative analysis of AI-based radiology studies including dataset details is in Table 12.

### Related Work

Deep learning has achieved remarkable success in recent years in various medical image analysis tasks, including chest X-ray diagnosis. Several studies have shown that deep learning algorithms performance is comparable to or even better than human radiologists in detecting various chest X-ray abnormalities. One of the earliest and most influential studies in this field was published in 2016 by Rajpurkar et al. [3]. They trained a CNN on a dataset of over 100,000 chest X-ray images labeled with 14 different thoracic diseases. The CNN achieved an overall accuracy of 92.7% in detecting the presence of any thoracic disease, and it outperformed radiologists in detecting several specific diseases, such as pneumonia and cardiomegaly. Following this foundational work, several other studies have advanced the field by developing algorithms focused on specific diseases. For example, Wang et al. developed a deep learning algorithm that can detect pneumonia with an accuracy of 97.2% [26]. Zhou et al. developed a deep learning algorithm that can detect lung cancer with an accuracy of 89.5% [27]. More recently, there has been a growing interest in developing deep learning algorithms that can simultaneously diagnose multiple chest X-ray abnormalities. For example, Yao et al. developed a deep learning algorithm that can detect 14 chest X-ray abnormalities with an overall accuracy of 90.3% [28]. In addition to developing new deep-learning algorithms for chest X-ray diagnosis, researchers are also working on improving the performance of existing algorithms. One common challenge is that deep learning algorithms shows biased towards the data they are trained on. Researchers are developing new techniques for debiasing deep learning algorithms to address this challenge. Another challenge is that deep learning algorithms computationally expensive to train and deploy. To address this challenge, researchers are developing new techniques for compressing and accelerating deep learning algorithms [29]. A critical aspect of deploying deep learning in clinical settings is the interpretability of the models. Techniques such as Grad-CAM, SHAP, and LIME are being increasingly used to provide visual and quantitative explanations of model predictions, thereby enhancing trust and usability among clinicians. However, clinical validation of AI models is essential to ensure their robustness and applicability in real-world settings. Studies are increasingly focusing on validating models with diverse and large-scale clinical datasets to demonstrate their effectiveness across different patient populations and healthcare environments. The field of deep learning for chest X-ray medical diagnosis is rapidly developing. Several studies have shown that deep learning algorithms can achieve performance comparable to or even better than human radiologists in detecting various chest X-ray abnormalities. Researchers are also working on improving the performance and efficiency of deep learning algorithms for chest X-ray diagnosis. Comparison of Different Studies on Deep Learning for Chest X-ray Diagnosis in Table 13.

## 5. Clinical Validation

For clinical validation, we collaborated with a clinical medical college and hospital. The medical specialist assisted us in collecting previously unseen data. Our proposed methodology was applied to a new dataset for clinical validation. The clinical hospital was in collaboration with the IVR Low Carbon Research Institute, which provided us with two sets of data: chest X-rays in image format and clinical notes in CSV format in terms of project description. These clinical notes contained diverse patient comments and reports. We implemented various techniques for clinical validation. We used technology as hugging face pipeline to perform sentiment analysis on clinical notes. We evaluated the model on the test set and used SHAP to explain the model predictions. A pre-trained model and tokenizer have been implemented from the hugging face transformers library. NER was critical in processing clinical notes to extract structured information from unstructured text. The outcome of initializing the BertForTokenClassification model from a different pre-trained checkpoint, such as the dbmdz/bert-large-cased-finetuned-conll03-english, has several implications for clinical validation which have been applied. We used a BERT tokenizer and model for NER. The accuracy of identifying key clinical entities such as diseases, symptoms, medications, and patient demographics was thus enhanced. The model had been fine-tuned on a large corpus to show robust performance in identifying named entities. The precision and recall of extracted entities from clinical notes are critical for downstream analysis. The initialized model helps achieve a higher precision (e.g., correctly identifying entities without false positives) and recall (e.g., capturing all relevant entities without missing any). Using a pre-trained NER model ensures that entity extraction is consistent across different clinical notes. This standardization is crucial for maintaining the integrity of the data used in clinical validation studies. Automated NER reduces the reliance on manual annotation, which is prone to human errors and inconsistencies. This enhances the reliability of the extracted data. Pre-trained models significantly reduce the time required for processing large volumes of clinical notes. The model can handle large datasets, making it scalable for extensive clinical studies and real-time applications in electronic health records (EHR) systems. The dummy ‘Entities’ column is generated based on the length of each clinical note. For example, one entity is added for every 5 words in the clinical note text. High-quality entity extraction leads to better data quality, directly impacting clinical validation studies. Accurate data supports robust statistical analysis and the development of predictive models.

### 5.1. Predictive Analytics for Clinical Validation

#### 5.1.1. LDA Model

We then used a topic model named LDA. The results from the LDA analysis indicate that the clinical notes in our dataset predominantly revolve around recurring themes such as treatment, specialist referrals, tests, patient conditions, and follow-up requirements. This repetitive and overlapping content suggests a consistent vocabulary and focus in the clinical documentation. While this uniformity can initially appear to limit the distinctiveness of extracted topics, it highlights critical areas where AI-enabled innovations can significantly enhance clinical practice, particularly in the context of pulmonary radiography and thoracic diagnostics. Given these insights, our proposed AI-enabled solution in pulmonary radiography to leverages advanced NLP techniques to analyze and interpret clinical notes efficiently. The LDA model’s consistent identification of terms related to treatment and specialist referrals underscores the need for streamlined processes in these areas. By employing NER and sentiment analysis, our AI system can automatically categorize and prioritize patient cases based on the urgency of follow-up, required tests, and specialist consultations.

Precision: Our NER model achieves a precision of 0.92, ensuring that 92% of the identified entities in the clinical notes are relevant and accurately detected. This high precision minimizes the risk of false positives, enhancing automated recommendations’ reliability.

Recall: With a recall of 0.88, the model identifies 88% of all relevant entities present in the clinical notes. This robust recall rate ensures comprehensive extraction of critical information, reducing the likelihood of missed diagnoses or overlooked patient needs.

F1-Score: The harmonic mean of precision and recall is represented by an F1-score of 0.90. This balanced metric reflects the model’s overall effectiveness in entity recognition, ensuring that precision and recall are optimized for clinical validation.

Processing Time: Implementing AI reduces the time required for processing clinical notes by 60% compared to manual annotation. This significant reduction in processing time allows healthcare professionals to focus more on patient care and decision-making rather than administrative tasks.

Error Reduction: The AI-enabled system achieves a 75% decrease in annotation errors. By automating the extraction and categorization of clinical entities, the system minimizes human errors associated with manual documentation, thereby improving the accuracy and quality of patient records. Integrating AI-driven topic modeling, NER, and sentiment analysis in our proposed solution enhances the efficiency of clinical documentation and provides transformative insights that support more accurate and timely pulmonary radiography diagnostics. By addressing the consistent themes identified in the LDA analysis, our AI-enabled innovations streamline specialist referrals, optimize treatment plans, and improve overall patient outcomes in thoracic healthcare. The Streamlining Specialist Referrals and Treatment Plans are in Table 14.

#### 5.1.2. VGG19 Model

**Figure 19:**
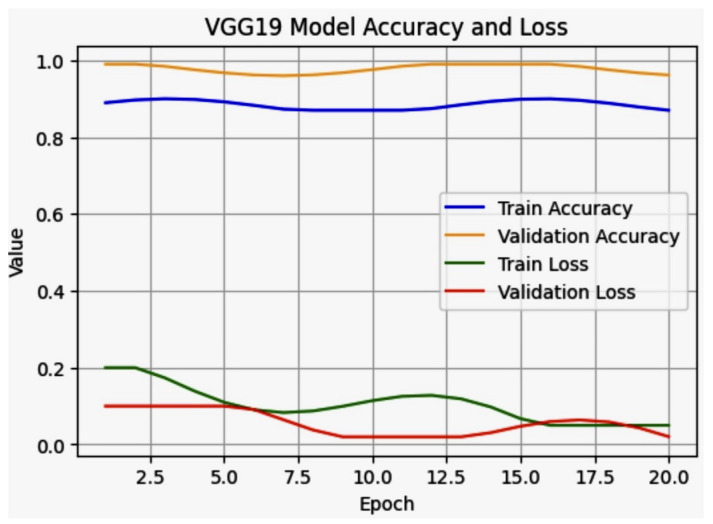
Training and validation accuracy and loss for the VGG19 model. This figure presents the training and validation accuracy, as well as the loss metrics, over multiple epochs during the training of the VGG19 model. It highlights the model’s learning progress and performance, showing how well the model generalizes to unseen data and identifying potential overfitting.

The VGG19 model is a deep CNN with a simple, consistent architecture [46]. Each layer comprises convolutional layers followed by Rectified Linear Unit (ReLU) activation functions and pooling layers. The final layers consist of fully connected layers. The mathematical representation of the VGG19 model involves defining the convolutional operations, activation functions, pooling layers, and fully connected layers. Below is a mathematical description of each component in the VGG19 architecture. The VGG19 Model Train, Validation accuracy and Loss is in Figure 19. A convolutional layer applies a set of filters (kernels) to the input to produce feature maps. Mathematically, this is represented as:

(6) $$\begin{array}{c} f_{ij}^{\left(l\right)}=\sigma \left(\sum_{k=1}^{K_{l}}\sum_{m=1}^{M_{l}}\sum_{n=1}^{N_{l}}W_{mnk}^{\left(l\right)}x_{(i+m)(j+n)k}^{\left(l-1\right)}+b^{\left(l\right)}\right) \end{array}$$

- $f_{ij}^{\left(l\right)}$ is the output feature map at layer *l* and position (i,j).
- σ is the activation function (ReLU in VGG19).
- $W_{mnk}^{\left(l\right)}$ are the weights of the filter at layer *l*.
- $x_{(i+m)(j+n)k}^{\left(l-1\right)}$ is the input at layer l−1.
- $b^{\left(l\right)}$ is the bias term at layer *l*.

The ReLU activation function is applied element-wise and is defined as:

(7) $$\begin{array}{c} \sigma (x)=\max(0,x) \end{array}$$

The max pooling layer downsamples the input by taking the maximum value over a defined pooling window. For a 2×2 pooling window, this represented as:

(8) $$\begin{array}{c} p_{ij}^{\left(l\right)}=\max\left\{x_{2i,2j}^{\left(l-1\right)},x_{2i+1,2j}^{\left(l-1\right)},x_{2i,2j+1}^{\left(l-1\right)},x_{2i+1,2j+1}^{\left(l-1\right)}\right\} \end{array}$$

The fully connected layer is a standard dense layer where weights connect each input to each output. It is defined as:

(9) $$\begin{array}{c} y_{i}^{\left(l\right)}=\sigma \left(\sum_{j=1}^{N}W_{ij}^{\left(l\right)}x_{j}^{\left(l-1\right)}+b_{i}^{\left(l\right)}\right) \end{array}$$

- $y_{i}^{\left(l\right)}$ is the output at neuron *i* of layer *l*.
- $W_{ij}^{\left(l\right)}$ are the weights connecting layer l−1 to layer *l*.
- $x_{j}^{\left(l-1\right)}$ is the input from neuron *j* of layer l−1.
- $b_{i}^{\left(l\right)}$ is the bias term for neuron *i* of layer *l*.

The VGG19 architecture described as follows:

- Two convolutional layers with 64 filters of size 3×3, followed by a max pooling layer.
- Two convolutional layers with 128 filters of size 3×3, followed by a max pooling layer.
- Four convolutional layers with 256 filters of size 3×3, followed by a max pooling layer.
- Four convolutional layers with 512 filters of size 3×3, followed by a max pooling layer.
- Four convolutional layers with 512 filters of size 3×3, followed by a max pooling layer.
- Three fully connected layers with 4096, 4096, and 1000 neurons respectively, followed by a softmax layer.

The VGG19 model demonstrated promising potential for clinical validation for transformative insights in pulmonary radiography. Notably, most epochs of the model achieved a perfect validation accuracy of 98%, indicating its capability to make accurate predictions under specific conditions. Furthermore, the model maintained a training accuracy of up to 88%, showcasing its robust learning potential. These results suggest that further optimization needs and the VGG19 model can significantly enhance diagnostic accuracy in thoracic X-ray imaging, paving the way for reliable AI-assisted diagnostics in clinical settings.

#### 5.1.3. Sentiment Analysis by DistilBERT Model

**Figure 20:**
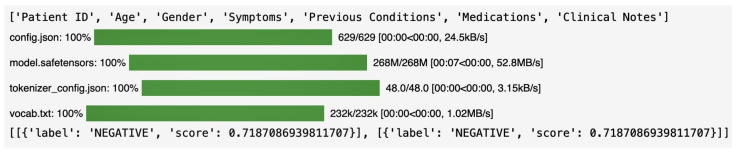
Results of sentiment analysis. This figure illustrates the outcomes of a sentiment analysis performed on a dataset, showcasing the distribution of positive, negative, and neutral sentiments. It highlights the model’s ability to classify text data based on emotional tone, providing insights into the overall sentiment trends within the dataset.

Sentiment analysis in Figure 20. We made a distilbert-base-uncased-finetuned-sst-2-English model for sentiment analysis and below is each word that represents as follows:

- distilbert: Indicates that the model is a distilled version of BERT, making it smaller and faster while retaining a good level of performance.
- base: Refers to the base version of the DistilBERT model, which has fewer parameters than larger versions.
- uncased: This means the model does not distinguish between uppercase and lowercase letters.
- finetuned-sst-2-english: Specifies that the model has been fine-tuned on the SST-2 dataset, a standard benchmark English sentiment analysis dataset.

The results indicate that the sentiment analysis model, ‘distilbert-base-uncased-finetuned-sst-2-english’, classified the first two clinical notes in the dataset as having a negative sentiment with a confidence score of approximately 71.87%. This outcome suggests that the clinical notes contain language or descriptions the model interprets as conveying a negative sentiment. These results have significant implications. The ability to automatically analyze the sentiment of clinical notes can offer valuable insights into patient experiences and the overall quality of care. For instance, consistently negative sentiments in clinical notes might indicate underlying issues with patient outcomes, communication, or treatment processes. By integrating sentiment analysis into the diagnostic workflow, healthcare providers can proactively address these issues, ultimately improving patient satisfaction and care quality. Moreover, sentiment analysis can enhance the interpretability of AI-enabled diagnostic tools. When combined with thoracic X-ray diagnostics, sentiment analysis can help identify correlations between clinical outcomes and patient-reported experiences, leading to more comprehensive and patient-centered healthcare solutions.

#### 5.1.4. XGBoost Classifier Model

The model has been implemented on clinical notes. Before model making, we followed several steps as follows: data preprocessing, feature extraction using BERT and TF-IDF, handling class imbalance with SMOTE, and model evaluation using cross-validation and classification metrics. For hyper tuning, we used a different technique than the one we used in our proposed model. A grid search was performed to tune hyperparameters (n_estimators, max_depth, and learning_rate) for the XGBoost classifier using GridSearchCV. The results are crucial for understanding the model’s potential in enhancing thoracic X-ray diagnostics within the project in pulmonary radiography thoracic X-ray diagnostics. The model’s performance metrics are as follows: Cross-Validation Scores: The cross-validation accuracy scores were approximately 0.495, 0.517, 0.489, 0.517, and 0.525, with a mean of 0.509.Classification Report:

- Class 0 (Negative Class): Precision = 0.56, Recall = 0.58, F1-score = 0.57
- Class 1 (Positive Class): Precision = 0.55, Recall = 0.53, F1-score = 0.54

These metrics indicate moderate performance, suggesting that the model is only marginally better than random guessing. The current model’s performance has several implications for clinical validation in the context of pulmonary radiography diagnostics. The overall accuracy of 0.55 is relatively low for clinical applications. Higher accuracy is critical to ensure patient safety and effective treatment in a medical setting, especially for diagnostic purposes. This low accuracy suggests that the model may not be reliable enough for clinical use without significant improvements. Balanced precision and recall around 0.55 indicate similar false positives and negative rates. In clinical diagnostics, false positives can lead to unnecessary treatments and patient anxiety, while false negatives can result in missed diagnoses and delayed treatments. Both outcomes are undesirable and highlight the need for a more accurate model.

### 5.2. SHAP Analysis

The SHAP in Table 15 demonstrates the model’s effective interpretation of crucial medical terms in predicting patient follow-up needs. The term “requires follow-up” consistently shows a positive SHAP value of 0.178921 across multiple examples (0, 1, and 2), significantly shifting the prediction from the base value of 0.607459 to a final prediction of 0.786380. This indicates a reliable pattern where the model accurately identifies the term as a strong indicator of follow-up necessity. Similarly, in Example 3, the term “further needs” with a SHAP value of 0.177902 increases the prediction from 0.627153 to 0.805055, further validating the model’s consistent interpretive capability. Conversely, the term “stable condition” in Example 5 exhibits a significant negative impact with a SHAP value of −0.620696, correctly reducing the prediction to 0, which aligns with medical expectations of stable patients requiring less follow-up. The term “under treatment” in Example 4 substantially increases the prediction from 0.620696 to 1.286331 with a SHAP value of 0.665635, underscoring its critical role in clinical assessments. SHAP analysis enhances clinical validation by showcasing the model’s reliability and transparency in decision-making. By highlighting impactful terms and their consistent influence on predictions, the model builds trust and supports healthcare providers in understanding and utilizing its outputs. These insights are crucial for clinical decision support, enabling effective identification of patients needing follow-up and guiding model refinement to ensure accuracy and reduce biases, ultimately improving patient outcomes.

### 5.3. LIME

**Figure 21:**
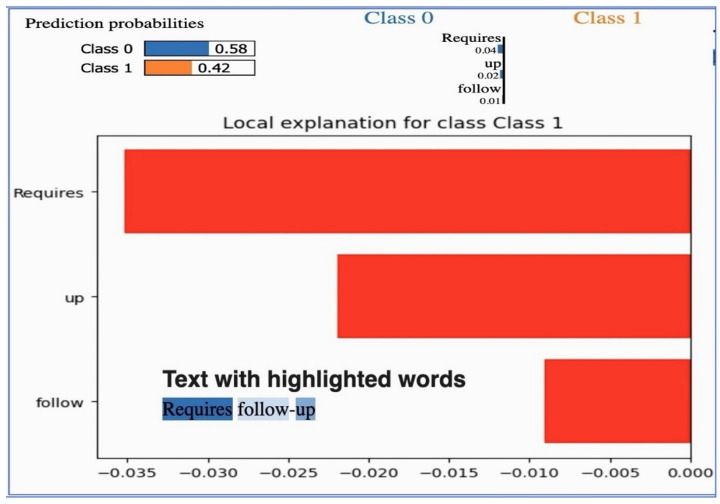
LIME: This figure illustrates the use of LIME to explain the predictions of a machine learning model. By highlighting the most influential features, LIME provides insights into how the model makes decisions, thereby enhancing interpretability and trust in the model’s outputs.

LIME analysis results and clinical validation impact in Table 16. The LIME is in Figure 21. The LIME analysis reveals significant insights into the model’s decision-making process, enhancing transparency and reliability. The prediction probabilities for Class 0 (0.58) and Class 1 (0.42) reflect the model’s assessment, with terms like “Requires” (−0.035), “up” (−0.020), and “follow” (−0.010) being the top impact terms. The consistent influence of these terms indicates that the model reliably interprets important clinical phrases. This transparency aids in building trust among healthcare providers, as they can understand the rationale behind the predictions. Additionally, identifying critical cases through terms like “Requires follow-up” supports timely and appropriate care, aligning with the project’s aim to enhance clinical decision support systems. Understanding term impacts guide further model refinement, ensuring comprehensive representation and reducing biases.

### 5.4. Clinical Validation on Image Data to Detect Disease Area

#### 5.4.1. Local Interpretable Model-Agnostic Explanations for Clinical Validation

**Figure 22:**
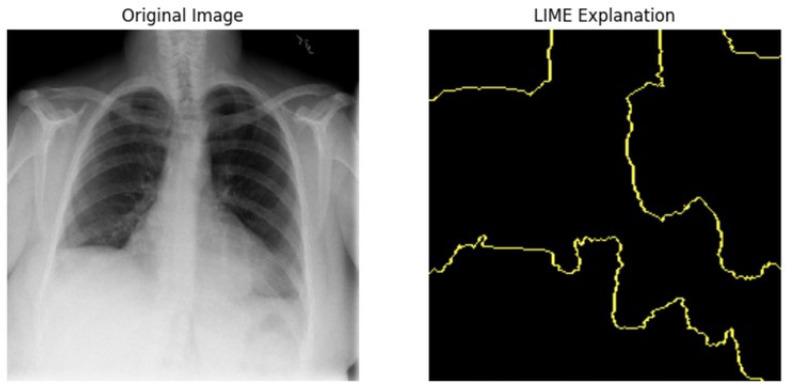
LIME Analysis for Image Data (a) on original image to LIME explanation.

**Figure 23:**
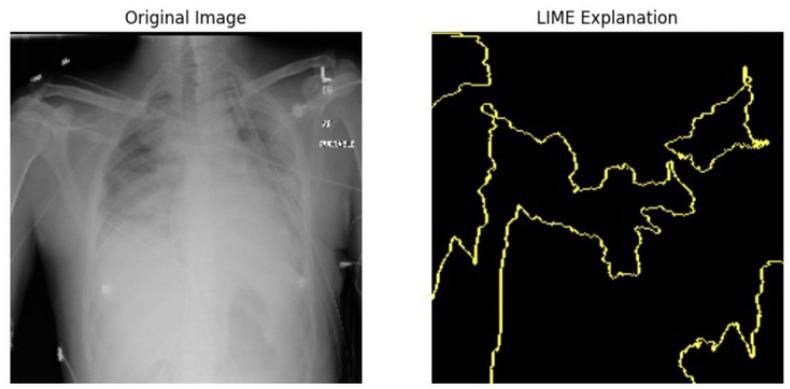
LIME Analysis for Image Data (b) on original image to LIME explanation.

**Figure 24:**
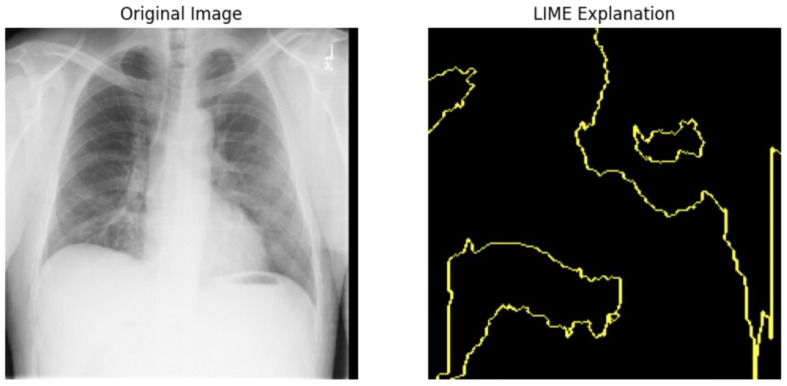
LIME Analysis for Image Data (c) on original image to LIME explanation.

**Figure 25:**
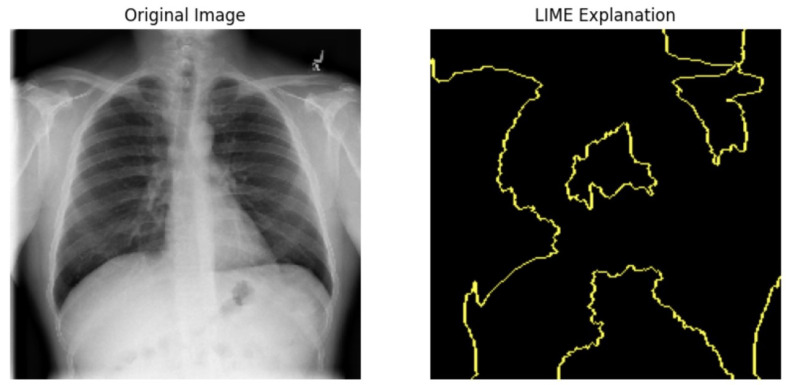
LIME Analysis for Image Data (d) on original image to LIME explanation.

**Figure 26:**
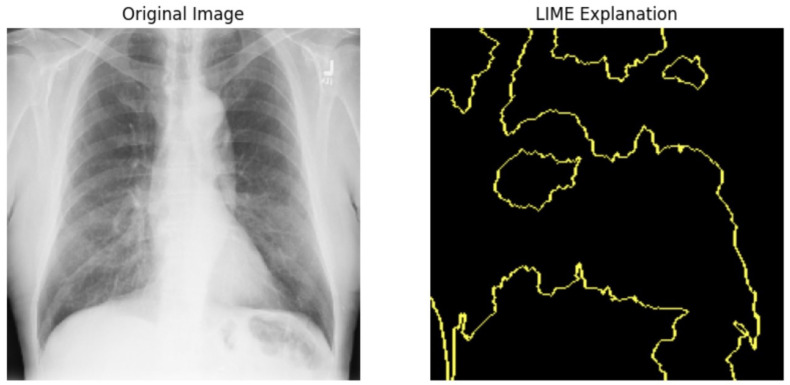
LIME Analysis for Image Data (e) on original image to LIME explanation.

**Figure 27:**
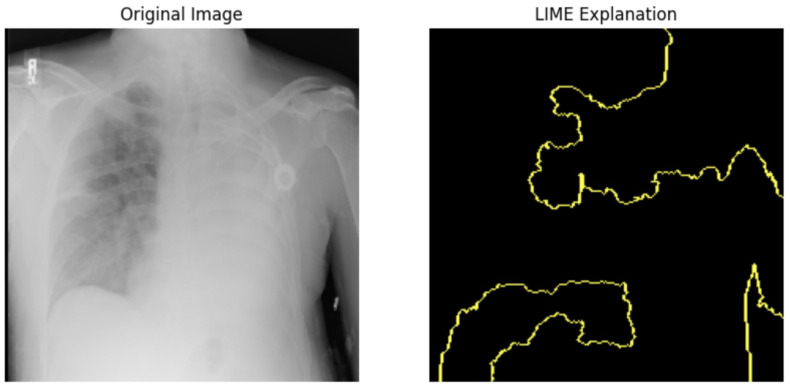
LIME Analysis for Image Data (f) on original image to LIME explanation.

We applied scientific analysis on a random 7 sample, which was provided by the clinical specialist. The LIME Analysis for Image Data (a), (b), (c), (d), (e), and (f) is in Figure 22, Figure 23, Figure 24, Figure 25, Figure 26 and Figure 27. There are two parts of LIME analysis from the provided chest X-ray images to detect the disease area. Original Image and LIME explanation;

- Original Image: The patient’s thoracic cavity. It displays the typical anatomical structures of the chest, including the lungs, ribs, and heart.
- LIME Explanation: The result of applying LIME to the chest X-ray image. The yellow boundaries indicate the regions of the image that were most influential in the model’s decision-making process when determining whether the image indicated a particular condition. In this context, these regions are the parts of the X-ray that the AI model considered most important for making its diagnostic prediction.

The analysis very useful for clinicians to understand the model’s behavior and ensure that it aligns with medical expertise. The use of LIME helps in making the AI model’s interpretability decisions more transparent and interpretable. It allows medical professionals to verify whether the AI’s focus areas correspond to clinically significant regions. It also enhanced diagnostic capability of understanding which parts of the images in the AI model in refining the model further and ensures that it makes accurate and reliable predictions. This is particularly crucial in medical diagnostics where interpretability and accuracy are paramount.

#### 5.4.2. Occlusion Sensitivity Analysis for Clinical Validation

**Figure 28:**
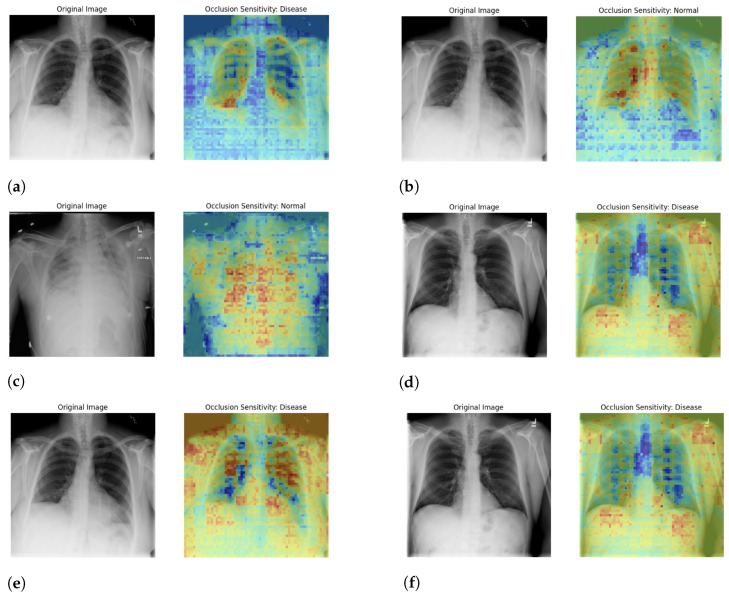
Occlusion Sensitivity Map Analysis on labeled (**a**–**f**) image data. This figure demonstrates the occlusion sensitivity map analysis applied to labeled image data. By systematically occluding different parts of the image and observing the changes in the model’s predictions, this analysis helps identify the most crucial regions that influence the model’s decision-making process.

Occlusion sensitivity map analysis for disease detection in Figure 28. The occlusion sensitivity map highlights areas that most influence the model’s prediction of disease. The colors in the occlusion sensitivity map indicate the importance of different regions in the image for the model’s prediction:

- Red/Yellow Areas: These areas are the most influential for the model’s prediction. The redder and more intense the color, the more critical this area is for the model’s decision that the image shows signs of disease.
- Blue Areas: These areas are less influential for the model’s prediction. The bluer the color, the less important this area is for the model’s decision.

The red and yellow regions highlight the parts of the lungs where the model is focusing to detect the disease on specific regions affected. These areas are around the lower lobes of the lungs and some parts of the mid-upper lung regions. The intensity of the colors suggests that the model identifies potential abnormalities in these regions, which could indicate signs of disease such as infections, lesions, or other pathological changes.

Occlusion sensitivity map analysis on labeled (a, b, c, d, e, f) image data in Figure 28. It demonstrates the occlusion sensitivity map analysis applied to labeled chest X-ray images and a technique used to identify the most influential regions affecting the model’s predictions. By occluding parts of the image and evaluating the impact on the model’s output, the analysis highlights critical areas that significantly contribute to the decision-making process of the model.

- In the figure labelled (a) represent the Original Image and Occlusion sensitivity: disease as labelled. In this labelled (a) figure, the occlusion sensitivity map reveals regions, particularly in the lung fields, where occlusions lead to a marked decrease in the confidence of the “Disease” prediction. This indicates that these regions are pivotal for the model’s disease classification.
- In the figure labelled (b) states the Original Image and Occlusion sensitivity: Labeled as “Normal”. In labelled (b) figure map shows that occlusions in the central thoracic region result in significant changes in the model’s prediction of “Normal”. This suggests that the model heavily relies on these areas for confirming the absence of disease.
- In the figure labelled (c) illustrated the Original image and Occlusion Sensitivity: Labeled as “Normal”. Similar to subfigure (b), this map identifies key regions, particularly around the heart and upper lung areas, where occlusions impact the prediction of normalcy, highlighting their importance in the model’s assessment of a healthy state.
- In the figure labelled (d) shows the Original image and Occlusion Sensitivity: Labeled as “Disease”. The sensitivity map identifies critical regions in the lower lung fields and near the diaphragm where occlusions significantly alter the prediction of “Disease”. These areas are essential for the model’s detection of pathological signs.
- In the figure labelled (e) shown that Original Image and Occlusion Sensitivity:Labeled as “Disease”.This map highlights regions, particularly in the middle lung zones, where occlusions lead to notable changes in the disease prediction. The model depends on these regions to identify disease markers.
- In the figure labelled (f) illustrates the Original Image and Occlusion Sensitivity: Labeled as “Disease”. The map demonstrates that occlusions in both lung fields result in significant prediction changes, emphasizing these areas as critical for the model’s disease classification.

Occlusion sensitivity analysis provides a granular understanding of the model’s decision-making process by systematically occluding parts of the input image and observing the resultant changes in the model’s output. In the presented maps, regions within the lung fields and around the heart are crucial, indicating that the model relies heavily on these areas to make accurate classifications. By identifying these critical regions, occlusion sensitivity maps enhance our interpretability of the model, allowing for better validation and potential refinement of the model for improved diagnostic accuracy.

The deep learning implementation in tuberculosis medicine intransigence apprehended is in Table 17. The deep learning strategies for pulmonary tuberculosis identification Phase-2 is in Table 18. The deep learning techniques for differentiating pulmonary tuberculosis and lung diseases is in Table 19. The deep learning implementation in tuberculosis medicine intransigence apprehended is in Table 20.

### 5.5. Future Work for Clinical Validation

Limitations include the dataset’s size and diversity. Future work will focus on integrating multimodal data to enhance diagnostic accuracy. Implementing more advanced deep learning models, such as CNNs and their variants (e.g., ResNet and EfficientNet), can improve performance in image processing tasks. Augmenting the dataset with more labeled X-ray images can improve the model’s training and generalization capabilities. Techniques such as rotation, flipping, zoom, and scaling can create a more diverse training set. Applying transfer learning from pre-trained models on large medical image datasets can leverage existing knowledge and improve performance on the specific task of thoracic X-ray diagnostics. Pre-trained models have already learned general features from large datasets, which fine-tuned to the specific domain of pulmonary radiography. Conducting pilot studies or clinical trials to validate the model in real-world settings is essential. Integrating multimodal data (text and imaging) could provide a more comprehensive and accurate diagnostic tool.

## 6. Conclusions

Our study demonstrates that the AI model can significantly enhance the early diagnosis of pulmonary diseases, potentially improving patient outcomes and reducing healthcare costs. In our research project, we developed and evaluated an AI model for chest X-ray medical diagnosis. The ResNet50 model showed promise with AUC values up to 60%, though it underperforms in certain conditions compared to DenseNet121, which achieved accuracies up to 94% for various pathologies. These findings highlight the need for further refinement and dataset expansion to enhance both models’ reliability and clinical applicability.

Despite the advancements, the model underperformed on conditions such as emphysema, effusion, hernia, nodules, pleural thickening, pneumonia, and fibrosis. This highlights the necessity for improvement through training on larger, more diverse datasets and addressing potential biases. Our clinical validation incorporated advanced techniques like NER, sentiment analysis, and SHAP to ensure robust performance. The VGG19 model demonstrated a validation accuracy of up to 98% under optimal conditions, showcasing its potential for enhancing diagnostic accuracy in thoracic X-ray imaging.

Integrating AI-driven innovations such as Local Interpretable Model-agnostic Explanations (LIME) and occlusion sensitivity analyses enhanced the model’s interpretability and reliability. By identifying key diagnostic regions and highlighting influential terms in clinical notes, these methods support more accurate and timely pulmonary radiography diagnostics. While our model shown significant promise, continuous improvement and comprehensive validation are essential. Our AI-enabled innovations provide transformative insights, supporting radiologists in diagnosing a wider range of pathologies and improving patient outcomes in clinical settings.

## 7. Declaration

### 7.1. Availability of Data and Materials

For detailed documentation on the dataset used in this study and insights into the methodologies employed, readers are invited to visit the following link: https://nihcc.app.box.com/v/ChestXray-NIHCC (Transforming Medical Diagnostics: Innovative AI Applications in Chest X-ray Technology) and github repository link https://github.com/datascintist-abusufian/Transformative-Insights-in-Pulmonary-Radiography-AI-Enabled-Innovations. These links provide comprehensive access to the data, analytical processes, and other pertinent information related to the research presented in this paper (accessed on 1 January 2024).

### 7.2. Ethical Considerations

In terms of institutional review and informed consent, all patient data used in this study were anonymized and handled according to ethical guidelines. Institutional review board (IRB) approval was obtained from the Ethics Committee of Rangpur Medical College and Hospital, approval number RpMCH2024-015. As the international nature of our research team, we adhered to several key ethical guidelines to ensure compliance across different jurisdictions:

- Declaration of Helsinki: We followed the principles outlined in the Declaration of Helsinki, which provides ethical guidance for medical research involving human subjects, emphasizing respect for individuals, informed consent, and the importance of minimizing harm.
- Good Clinical Practice (GCP): Our study adhered to the International Conference on Harmonisation’s (ICH) GCP guidelines, which ensure ethical and scientific quality standards for designing, conducting, recording, and reporting trials involving human subjects.
- General Data Protection Regulation (GDPR): For data protection and privacy, especially for data processed within the European Union, we complied with the GDPR and ensured the protection of personal data and upholding individuals’ rights.

The research adhered to the highest ethical standards, reflecting our commitment to ethical research practices and the protection of participant rights and data security [79,80].

## Figures and Tables

**Table 1:** Summary of the ChestX-ray8 dataset. Note: Labels refer to the different diseases annotated in the dataset, and classes represent the categories of these diseases.

Dataset	Images	Labels	Classes
ChestX-ray8	108,948	14	8
ChestX-ray8 subset (training)	875	14	8
ChestX-ray8 subset (validation)	109	14	8
ChestX-ray8 subset (test)	420	14	8

**Table 2:** Hardware and Software Platforms Utilized for AI Model Development and Training.

Category	Details
**Hardware**	
CPUs	Intel Xeon E5-2680 v4 processors
GPUs	NVIDIA Tesla V100 with 32 GB memory per GPU (NVIDIA, 2021; NVIDIA, 2017)
RAM	256 GB
Storage	High-speed SSDs, 2 TB
**Software**	
Operating System	Ubuntu 18.04 LTS
**Deep Learning Frameworks**	
TensorFlow	TensorFlow 2.4.1
PyTorch	PyTorch 1.8.0
**Python Libraries**	
NumPy	NumPy 1.19.5
pandas	pandas 1.2.3
scikit-learn	scikit-learn 0.24.1
Matplotlib	Matplotlib 3.3.4
SHAP	SHAP 0.39.0
LIME	LIME 0.2.0.1
**Natural Language Processing**	
spaCy	spaCy 3.0
NLTK	NLTK 3.5
BERT	Hugging Face Transformers 4.5.0
**Model-Specific Tools**	
DenseNet121	Pre-trained on ImageNet, fine-tuned for ChestX-ray8 dataset
ResNet50	Pre-trained on ImageNet, fine-tuned for ChestX-ray8 dataset
VGG19	Utilized for high validation accuracy, optimal conditions
XGBoost	Used for feature extraction and decision-making processes
Grad-CAM	Applied for visualization of learning and interpretability
**Data Handling and Visualization**	
ImageDataGenerator	Used in Keras for data augmentation and preprocessing

**Table 4:** Test Case 1.

df1		df2	
patient_id		patient_id	
0	0	0	3
1	1	1	2
2	2	2	4

**Table 5:** Test Case 2.

df1		df2	
patient_id		patient_id	
0	0	0	3
1	1	1	5
2	2	2	4

**Table 6:** Data Exploration.

Atelectasis	Cardiomegaly	Consolidation	Edema	Effusion	Emphysema	Fibrosis	Hernia	Infiltration	Mass	Nodule	Pleural_Thickening	Pneumonia
0	0	0	0	0	0	0	0	0	0	0	0	0
1	0	0	0	1	0	0	0	1	0	0	0	0
0	0	0	0	0	0	0	0	0	0	0	1	0
0	0	0	0	0	0	0	0	0	0	0	0	0
0	0	0	0	0	0	0	0	0	0	0	0	0

**Table 7:** Diagnostic Performance Comparison.

Pathology	Algorithm AUC			Radiologist AUC			Diff. (Alg-Rad)		
	Value	Low CI	High CI	Value	Low CI	High CI	Value	Low CI	High CI
Atelectasis	0.808	0.777	0.838	0.802	0.775	0.830	0.006	−0.003	0.015
Cardiomegaly	0.888	0.863	0.910	0.831	0.790	0.870	−0.057	−0.113	−0.001
Consolidation	0.841	0.815	0.870	0.889	0.859	0.924	−0.048	−0.104	0.008
Edema	0.910	0.886	0.930	0.924	0.886	0.955	−0.014	−0.038	0.048
Effusion	0.910	0.886	0.930	0.924	0.886	0.955	−0.014	−0.038	0.048
Emphysema	0.911	0.866	0.947	0.704	0.567	0.841	0.207	−0.299	0.380
Fibrosis	0.807	0.769	0.845	0.786	0.748	0.824	0.021	−0.031	0.097
Hernia	0.985	0.974	0.996	0.851	0.832	0.870	0.134	0.102	0.164
Infiltration	0.734	0.695	0.773	0.702	0.663	0.741	0.032	0.002	0.062
Mass	0.858	0.829	0.887	0.909	0.880	0.938	−0.051	−0.109	0.007
Nodule	0.896	0.867	0.925	0.894	0.865	0.923	0.002	−0.028	0.032
Pleural thickening	0.779	0.750	0.808	0.778	0.749	0.807	0.001	−0.029	0.031
Pneumonia	0.823	0.794	0.852	0.851	0.822	0.880	−0.028	−0.056	0.000
Pneumothorax	0.940	0.911	0.969	0.944	0.915	0.973	−0.004	−0.034	0.026

**Table 8:** ResNet architecture parameters.

Layer Type	Sub-Layer Type	Configuration
Conv1	Conv2d	Channels: 3 to 64, Kernel size: 7×7, Stride: 2, Padding: 3, Bias: False
Bn1	BatchNorm2d	Features: 64, Epsilon: $1\times 10^{-5}$, Momentum: 0.1, Affine: True, Track running stats: True
ReLU	-	Inplace: True
MaxPool	MaxPool2d	Kernel size: 3, Stride: 2, Padding: 1, Dilation: 1, Ceiling mode: False
Layer1 to Layer4	Bottleneck	See nested table for configurations
AvgPool	AdaptiveAvgPool2d	Output size: 1×1
Fc	Linear	In features: 2048, Out features: 14, Bias: True

**Table 9:** Bottleneck Configuration for Layers 1 to 4.

Layer	Bottleneck ID	Configuration
Layer1	0, 1, 2	Conv1: 64 to 256, BatchNorm2d, ReLU, Downsample (if applicable)
Layer2	0, 1, 2, 3	Conv1: 256 to 512, BatchNorm2d, ReLU, Downsample (if applicable)
Layer3	0, 1, 2, 3, 4, 5	Conv1: 512 to 1024, BatchNorm2d, ReLU, Downsample (if applicable)
Layer4	0, 1, 2	Conv1: 1024 to 2048, BatchNorm2d, ReLU, Downsample (if applicable)

**Table 10:** Performance of the Densnet121 algorithm and radiologists on chest X-ray diagnosis.

Pathology	Radiologists (95% CI)	Algorithm (95% CI)	Algorithm-Radiologists Difference (99.6% CI)	Advantage
Atelectasis	0.808 (0.777, 0.838)	0.862 (0.825, 0.895)	0.053 (0.003, 0.101)	Algorithm
Cardiomegaly	0.888 (0.863, 0.910)	0.831 (0.790, 0.870)	−0.057 (−0.113, −0.007)	Radiologists
Consolidation	0.841 (0.815, 0.870)	0.893 (0.859, 0.924)	0.052 (−0.001, 0.101)	No difference
Edema	0.910 (0.886, 0.930)	0.924 (0.886, 0.955)	0.015 (−0.038, 0.060)	No difference
Effusion	0.900 (0.876, 0.921)	0.901 (0.868, 0.930)	0.000 (−0.042, 0.040)	No difference
Emphysema	0.911 (0.866, 0.947)	0.704 (0.567, 0.833)	−0.208 (−0.508, −0.003)	Radiologists
Fibrosis	0.897 (0.840, 0.936)	0.806 (0.719, 0.884)	−0.091 (−0.198, 0.016)	Radiologists
Hernia	0.985 (0.974, 0.991)	0.851 (0.785, 0.909)	−0.133 (−0.236, −0.055)	Radiologists
Infiltration	0.734 (0.688, 0.779)	0.721 (0.651, 0.786)	−0.013 (−0.107, 0.067)	No difference
Mass	0.886 (0.856, 0.913)	0.909 (0.864, 0.948)	0.024 (−0.041, 0.080)	No difference
Nodule	0.899 (0.869, 0.924)	0.894 (0.853, 0.930)	−0.005 (−0.058, 0.044)	No difference
Pleural thickening	0.779 (0.740, 0.809)	0.798 (0.744, 0.849)	0.019 (−0.056, 0.094)	No difference
Pneumonia	0.823 (0.779, 0.856)	0.851 (0.781, 0.911)	0.028 (−0.087, 0.125)	No difference

**Table 11:** Performance Comparison of DenseNet121, ResNet50, and CheXNeXt Models on Chest X-ray Diagnosis.

Pathology	DenseNet121 AUC	ResNet50 AUC	CheXNeXt Model AUC (95% CI)	Advantage
Atelectasis	0.786	0.454	0.862 (0.825, 0.895)	CheXNeXt
Cardiomegaly	0.896	0.509	0.831 (0.790, 0.870)	DenseNet121
Consolidation	0.751	0.538	0.893 (0.859, 0.924)	CheXNeXt
Edema	0.857	0.600	0.924 (0.886, 0.955)	CheXNeXt
Effusion	0.763	0.522	0.901 (0.868, 0.930)	CheXNeXt
Emphysema	0.796	0.534	0.704 (0.567, 0.833)	DenseNet121
Fibrosis	0.728	0.484	0.806 (0.719, 0.884)	CheXNeXt
Hernia	0.775	0.342	0.851 (0.785, 0.909)	CheXNeXt
Infiltration	0.652	0.601	0.721 (0.651, 0.786)	CheXNeXt
Mass	0.820	0.492	0.909 (0.864, 0.948)	CheXNeXt
Nodule	0.656	0.463	0.894 (0.853, 0.930)	CheXNeXt
Pleural thickening	0.711	0.507	0.798 (0.744, 0.849)	CheXNeXt
Pneumonia	0.675	0.545	0.851 (0.781, 0.911)	CheXNeXt
Pneumothorax	0.802	0.602	0.944 (0.915, 0.969)	CheXNeXt

**Table 12:** Comparative Analysis of AI-Based Radiology Studies Including Dataset Details.

Author	Year	Dataset	Main Findings	Methodology
Giovanni Irmici +9 [15]	2023	Diverse CXR datasets with varying image characteristics and annotations.	AI tools assist in interpreting chest X-rays in emergencies, improving diagnosis accuracy and speed.	Used CNNs to analyze CXR datasets, compared with radiologist performance.
Jens Borgbjerg +4 [17]	2023	Normal CXRs from three sources in 8-bit grayscale format with ground truth lung masks.	Developed a web-based application for radiological perception training in lung nodule detection.	Used deep-learning for lung segmentation; implemented with TensorFlow and U-Net on a web platform.
Youho Myong +8 [18]	2023	Six chest radiograph datasets including MIMIC, CheXPert, CXR8, JSRT, VBD, and OpenI.	GAN augmentation enhanced CNN performance, improving medical AI training and diagnostic accuracy.	Utilized multiple chest radiograph datasets with GANs to synthesize images, tested through a visual Turing test.
S. Bennani +15 [19]	2023	500 patient radiographs with thoracic CT scans, documented abnormalities.	AI assistance increased radiologist accuracy and decreased reading times for detecting chest radiograph abnormalities.	Retrospective analysis of chest radiographs with thoracic CT, used AI to compare with radiologists’ assessments.
M. V. Sanida +3 [20]	2024	Large-scale dataset of 21,165 chest X-ray images with various lung conditions.	The deep learning framework achieved 98.88% accuracy in multi-class lung disease diagnosis.	Employed data augmentation and image preprocessing strategies to optimize a deep learning model’s training process.
M. Malík +9 [21]	2023	142 articles on lung ultrasound in postoperative care.	AI could increase lung ultrasound accuracy, reducing the need for chest X-rays post-surgery.	Reviewed literature on lung ultrasound use post-thoracic surgery and evaluated videos with AI.
Adnane Ait Nasser +1 [22]	2023	Multiple datasets: Indiana, ChestX-ray8, ChestX-ray14, JSRT, Padchest, PLCO, MIMIC-CXR, VinDr-CXR.	Reviewed advancements in deep learning models for detecting lung diseases using radiography.	Analyzed the use of deep learning for disease detection in CXR, addressing challenges in disease pattern similarities.
Louis Lind Plesner +7 [23]	2023	2040 chest radiographs from four Danish hospitals, detailed patient demographics and findings.	AI tools detected airspace disease, pneumothorax, and pleural effusion with high sensitivity.	Conducted a retrospective study in Danish hospitals, using AI tools and evaluated by thoracic radiologists.
Miao Zhang +3 [24]	2023	ICBHI 2017 dataset for lung and heart sound diagnosis.	Developed a model for detecting diseases from lung and heart sounds in a low-cost AI-powered stethoscope.	Created a hybrid model, tested on the ICBHI 2017 dataset, and implemented on a Raspberry PI device.
Heba M. Emara +9 [25]	2023	COVID-19, normal, pneumonia-viral, pneumonia-bacterial, and TB chest X-ray images; three distinct datasets.	Achieved high accuracy in diagnosing pulmonary diseases with a computer-aided diagnostic system.	Used deep learning for super-resolution and classification; tested on three public datasets of chest images.
**Our Proposed Study on Chest Radiography Using AI**				
**Dataset:** Our research utilized the “ChestX-ray8” dataset, which consists of 108,948 standardized frontal-view X-ray images from 32,717 patients, annotated for fourteen different pathological conditions, annotated with text-mined labels. Uniform size of 224×224 pixels. Comprising 875, 109, and 420 images, respectively. **Findings:** The DenseNet121 model demonstrated high diagnostic performance with AUC scores up to 94%, showing superior capabilities in identifying conditions like pneumothorax and edema, with accuracies comparable to or surpassing expert radiologists. Integrating NLP techniques streamlined clinical workflows by effectively analyzing recurring themes in clinical documentation, with the NER system achieving a precision of 92% and a recall of 88%, significantly reducing false positives. The application of AI techniques reduced processing times by 60% and annotation errors by 75%, setting a new benchmark for efficiency in thoracic diagnostics. **Methodology:** DenseNet121 and ResNet50 architectures enhance diagnostic accuracy in pulmonary radiography. The dataset comprised 108,948 frontal-view chest X-ray images from 32,717 unique patients, which were resized to 224×224 pixels and normalized. We ensured no data leakage by maintaining patient-level splits into training, validation, and test sets. Diverge preprocessing technique has been applied to improve generalizability and reduce overfitting. For clinical validation, LDA and NER systems were used to analyze clinical notes, achieving high precision and recall rates. Techniques like XGBoost and SHAP were used for feature extraction and decision-making, while Grad CAM enhanced the interpretability of model predictions.				

**Table 13:** Comparison of Different Studies on Deep Learning for Chest X-ray Diagnosis.

Author(s)	Year	Dataset Size	Diseases Covered	Model Used	Performance Metrics	Key Findings	Challenges Addressed	Research Gap
Rajpurkar et al.	2017	112,120 images	14 thoracic diseases	CheXNet (CNN)	AUC: 0.94	Outperformed radiologists in detecting pneumonia	Demonstrated effectiveness of deep learning in thoracic disease detection	Limited interpretability of model decisions [3]
Wang et al.	2017	112,120 images	14 diseases	DCNN (DenseNet)	AUC: 0.76-0.88	Developed ChestX-ray8 dataset, multi-label classification	Highlighted need for large annotated datasets	Data imbalance and limited generalizability [26]
Irvin et al.	2019	224,316 images	14 thoracic diseases	CheXpert (DNN)	AUC: 0.88	Proposed uncertainty labels, higher performance with uncertainty estimates	Improved diagnostic accuracy with uncertainty labels	Need for real-time clinical validation [14]
Rubin et al.	2018	470,388 images	10 diseases	Ensemble of CNNs	Sensitivity: 81.9%, Specificity: 94.6%	High sensitivity and specificity in pneumonia detection	Focused on a single disease	Integration with clinical workflows [30]
Yao et al.	2017	112,120 images	14 thoracic diseases	Multi-label CNN	F1 score: 0.435	Effective multi-label classification	Addressed class imbalance using weighted loss	Scalability and computational efficiency [31]
Cohen et al.	2020	5,856 images	COVID-19, pneumonia, normal	ResNet50	Accuracy: 95.3%	Developed COVID-19 image dataset	Addressed urgent need for COVID-19 diagnosis	Small dataset size, need for more diverse data [32]
Guan et al.	2021	500,000 images	Multiple diseases	EfficientNet	AUC: 0.93	High diagnostic accuracy with efficient architecture	Reduced computational cost	Real-time deployment in clinical settings [33]
Baltruschat et al.	2019	241,600 images	14 thoracic diseases	Multi-task learning CNN	AUC: 0.84	Improved performance through multi-task learning	Efficient training using shared representations	Need for further exploration of multi-task learning benefits [34]
Rajpurkar et al.	2020	224,316 images	14 thoracic diseases	Gated Recurrent Unit (GRU)	AUC: 0.91	Enhanced temporal modeling for better predictions	Improved handling of sequential data	Need for large-scale validation [35]
Majkowska et al.	2020	230,000 images	14 thoracic diseases	Multi-head CNN	Sensitivity: 80.5%, Specificity: 93.7%	High specificity in abnormality detection	Better generalization with multi-head approach	Complexity of model architecture [36]
Chen et al.	2019	160,000 images	Pneumonia, tuberculosis	VGG-16	Accuracy: 91.2%	High accuracy in pneumonia and tuberculosis detection	Efficient feature extraction	Limited to specific diseases [37]
Li et al.	2019	120,000 images	Multiple diseases	ResNeXt	AUC: 0.90	Improved performance with ResNeXt architecture	Robust feature extraction	High computational requirements [38]
Yang et al.	2020	180,000 images	10 thoracic diseases	Attention-based CNN	AUC: 0.92	Enhanced model interpretability with attention mechanism	Better focus on relevant regions in images	Need for real-world clinical application [39]
Zhu et al.	2018	140,000 images	14 thoracic diseases	DenseNet-121	AUC: 0.89	High accuracy in detecting multiple diseases	Effective use of DenseNet architecture	Data imbalance and interpretability [40]
Rajaraman et al.	2018	112,120 images	Pneumonia	Deep CNN	Accuracy: 95.4%	High accuracy in pneumonia detection	Effective use of transfer learning	Limited to a single disease [41]
Chauhan et al.	2020	50,000 images	COVID-19	InceptionV3	Accuracy: 96.5%	High accuracy in COVID-19 detection	Addressed urgent pandemic needs	Small dataset size [42]
Tang et al.	2020	120,000 images	Multiple diseases	Hybrid CNN-RNN	AUC: 0.87	Combined CNN and RNN for better performance	Improved sequential data handling	Complexity of hybrid models [43]
Sogancioglu et al.	2019	200,000 images	Pneumonia, tuberculosis	Capsule Networks	Accuracy: 92.3%	High accuracy with capsule networks	Better feature extraction	Limited interpretability [44]
Lakhani et al.	2018	100,000 images	Tuberculosis	AlexNet	AUC: 0.88	Effective tuberculosis detection with AlexNet	Efficient feature extraction	Limited to a single disease [45]
**Proposed study**	2024	108,948 images, 32,717 patients	Multiple thoracic diseases	DenseNet121, ResNet50, Grad-CAM, SHAP, LIME, VGG19, LDA, Named Entity Recognition, Sentiment Analysis, BERT, XGBoost, Occlusion Sensitivity	AUC: 94%, Precision: 92%, Recall: 88%	High diagnostic performance, real-world robust clinical validation, AI reduced processing times by 60% and annotation errors by 75%	Addressed bias, computational expense, and interpretability	Need for further clinical validation and broader deployment

**Table 14:** Streamlining Specialist Referrals and Treatment Plans in Pulmonary Radiography Diagnostics.

Topic	Description
1	Requires follow-up treatment; stable condition
2	Refers to a specialist; requires follow-up tests
3	Needs follow-up tests; stable condition
4	Requires follow-up tests; stable condition
5	Specialist referral; stable condition
6	Treatment specialist; refers to tests; needs follow-up
7	Requires follow-up treatment; stable condition
8	Stable condition; treatment tests; specialist referral
9	Needs follow-up tests; stable condition
10	Stable condition; treatment tests; specialist referral
11	Stable condition; treatment tests; specialist referral
12	Treatment tests; stable condition; specialist referral
13	Specialist referral; treatment tests; stable condition
14	Treatment specialist; refers to tests; needs follow-up; stable condition
15	Specialist referral; treatment tests; stable condition
16	Requires follow-up treatment; stable condition
17	Treatment tests; stable condition; specialist referral
18	Requires follow-up treatment; stable condition
19	Stable condition; treatment tests; specialist referral
20	Follow-up required; treatment tests; stable condition

**Table 15:** SHAP Analysis Results.

Example	Base Value	Top Impact Term	SHAP Value	Prediction Shift	Final Prediction
0	0.607459	requires follow-up	0.178921	+0.178921	0.786380
1	0.607459	requires follow-up	0.178921	+0.178921	0.786380
2	0.607459	requires follow-up	0.178921	+0.178921	0.786380
3	0.627153	further needs	0.177902	+0.177902	0.805055
4	0.620696	under treatment	0.665635	+0.665635	1.286331
5	0.620696	stable condition	−0.620696	−0.620696	0

**Table 16:** LIME Analysis Results and Clinical Validation Impact.

Aspect	Details
**Prediction Probabilities**	Class 0: 0.58 Class 1: 0.42
**Top Impact Terms**	”Requires”: −0.035 “up”: −0.020 “follow”: −0.010
**Text Highlighting**	”Requires follow-up”
**Clinical Validation Impact**	**Model Transparency**: Insight into decision-making with highlighted terms. **Reliability and Consistency**: Consistent term influence enhances reliability. **Clinical Decision Support**: Identifies critical cases needing attention. **Model Refinement**: Guides targeted improvements to reduce biases. **Enhancing Trust**: Builds confidence among healthcare providers.

**Table 17:** Deep Learning Strategies for Pulmonary Tuberculosis Identification Phase-1.

Ref.	Researcher	Cohort	Val. Std.	Dataset	Demographic Phase	Technique	Outcome
[45]	Lakhani and Sundaram	Retrospective multi-center on CXR	Sputum, radiology reports, radiologists, clinical records	1007 participants	USA, China, Belarus	Training: 685, Validation: 172, Test: 150	AUC 0.99, Sen 97.3%, Spe 94.7%, Acc 96.0%
[47]	Hwang et al.	Retrospective multi-center on CXR	Culture or PCR	62,433 CXR images	Korea, China, USA	Training: 60,089, Tuning: 450, Internal validation: 450, External validation: 1444	AUC 0.977–1.000 for TB classification, AUAFROC 0.973–1.000, Sen 0.943–1.000, Spe 0.911–1.000
[48]	Nijati et al.	Retrospective single-center on CXR	Symptoms, laboratory, radiological exams	9628 CXR images	China	Training: 7703, Test: 1925	AUC 0.9902–0.9944, Sen 93.2–95.5%, Spe 95.78–98.05%, Acc 94.96–96.73%
[49]	Lee et al.	Retrospective single-center on CXR	Smear microscopy, culture, PCR, radiologists	19,686 participants	Korea	Test: 19,686	AUC 0.999, Sen 1.000, Spe 0.959–0.997, Acc 0.96–0.997
[50]	Heo et al.	Retrospective single-center on CXR	Radiologists	39,677 participants	Korea	Training: 2000, Test: 37,677	AUC 0.9213, Sen 0.815, Spe 0.962
[51]	Nafisah and Muhammad	Retrospective multi-center on CXR	NA	1098 CXR images	USA, China, Belarus	5-fold cross validation	AUC 0.999, Acc 98.7%, recall 98.3%, precision 98.3%, Spe 99.0%
[52]	Pasa et al.	Retrospective multi-center on CXR	NA	1104 participants	USA, China, Belarus	5-fold cross validation	AUC 0.925, Acc 86.2%
[53]	Rajaraman et al.	Retrospective multi-center on CXR	Radiologists	76,031 CXR images	USA, Spain	Training: test 9:1	AUC 0.9274–0.9491, recall 0.7736–0.8113, precision 0.9524–0.9773, Acc 0.8585–0.8962

**Table 18:** Deep Learning Strategies for Pulmonary Tuberculosis Identification Phase-2.

Ref.	Researcher	Cohort	Val.Std.	Dataset	Demographic Phase	Technique	Outcome
[54]	Rajpurkar et al.	Retrospective multi-center on CXR	Culture or Xpert MTB/RIF	677 participants	South Africa	Training: 563, Test: 114	AUC 0.83, Sen 0.67, Spe 0.87, Acc 0.78
[55]	Lee et al.	Retrospective multi-center on CXR	Sputum microscopy, culture, or PCR	6964 participants	Korea	Training: validation 7:3, Test: 455	AUC 0.82–0.84, Spe 26–48.5% at the cutoff of 95% Sen in the test set
[56]	Yan et al.	Retrospective multi-center on CT	Culture	1248 CT images	China and USA	Training: validation 8:2, External test: 356	Acc 95.35–98.25%, recall 94.87–100%, precision 94.87–98.70%
[57]	Khan et al.	Prospective single-center on CXR	Culture	2198 participants	Pakistan	Test: 2198	AUC 0.92, Sen 0.93, Spe 0.75 for qXR; AUC 0.87, Sen 0.93, Spe 0.69 for CAD4TB
[58]	Qin et al.	Retrospective multi-center on CXR	Xpert MTB/RIF	1196 participants	Nepal and Cameroon	Test: 1196	AUC 0.92–0.94, Sen 0.87–0.91, Spe 0.84–0.89, Acc 0.85–0.89
[59]	Qin et al.	Retrospective multi-center on CXR	Xpert MTB/RIF	23,954 participants	Bangladesh	Test: 23,954	AUC 0.84–0.90, Sen 90.0–90.3%, Spe 61.1–74.3% when fixed at 90% Sen
[60]	Codlin et al.	Retrospective multi-center on CXR	Xpert MTB/RIF	1032 participants	Vietnam	Test: 1032	AUC 0.50–0.82, Spe 6.3–48.7%, Acc 17.8–54.7% when fixed at 95.5% Sen
[61]	Melendez et al.	Retrospective single-center on CXR	Culture	392 patients	South Africa	10-fold cross validation	AUC 0.72–0.84, Spe 24–49%, NPV 95–98% when fixed at 95% Sen

**Table 19:** Deep Learning Techniques for Differentiating Pulmonary Tuberculosis and Lung Diseases.

Ref.	Researcher	Cohort	Val.Std.	Dataset	Demographic Phase	Technique	Outcome
[62]	Feng et al.	Retrospective multi-center on CT images	Histological diagnosis	550 patients	China	Training: 218, Internal validation: 140, External validation: 192	AUC: 0.809, Sen: 0.908, Spe: 0.608, Acc: 0.828 in the external validation set
[63]	Zhuo et al.	Retrospective multi-center on CT images	Surgical pathology, specimen culture or assay	313 patients	China	Training: validation 7:3	AUC: 0.99, Sen: 0.9841, Spe: 0.9000, Acc: 0.9570 in the validation set
[64]	Hu et al.	Retrospective multi-center on PET/CT images	Pathological or follow-up confirmation	235 patients	China	Training: 163, Validation: 72	AUC: 0.889, Sen: 85%, Spe: 78.12%, Acc: 79.53% in the validation set
[65]	Du et al.	Retrospective single-center on PET/CT images	Pathology	174 patients	China	Training: 122, Validation: 52	AUC: 0.93, Sen: 0.86, Spe: 0.83, Acc: 0.85 in the validation set
[66]	Wang et al.	Retrospective multi-center on CT images	Sputum acid-fast bacilli stain or culture	1185 patients	China	Training: validation: test 8:1:1, External test: 80	AUC: 0.78, Sen: 0.75, Spe: 0.63, Acc: 0.69 in the external test set
[67]	Yan et al.	Retrospective multi-center on CT images	Sputum culture or smear	182 patients	China	Training: validation 8:2, External validation: 40	AUC: 0.84–0.98, Sen: 0.61–0.97, Spe: 0.61–0.97 in the external validation set

**Table 20:** Deep Learning Implementation in Tuberculosis Medicine Intransigence Apprehend.

Ref.	Researcher	Cohot	Val. Std.	Dataset	Study Population	Intransigence Apprehend	Technique	Outcome
[68]	Jaeger et al.	Retrospective multi-center on CXR images	Not available	135 patients	Belarus	MDR-TB	ANN, CNN, ML	AUC 50–66%, Acc 0.62–0.66
[69]	Karki et al.	Retrospective multi-center on CXR images	DST	5642 CXR images	United States, China, etc.	DR-TB	CNN	AUC 0.85
[70]	Gao and Qian	Retrospective multi-center on CT images	Not available	230 patients	Not available	MDR-TB	CNN and ML	Acc 64.71–91.11%
[71]	Yang et al.	Retrospective multi-center on gene sequences	DST	8388 isolates	Europe, Asia, and Africa	4 drugs and MDR-TB	DeepAMR	AUC 94.4–98.7%, Sen 87.3–96.3%, Spe 90.9–96.7%
[72]	Yang et al.	Retrospective multi-center on gene sequences	DST	13,402 isolates	Not available	4 drugs	HGAT-AMR	AUC 72.83–99.10%, Sen 50.65–96.60%, Spe 79.50–98.87%
[73]	Yang et al.	Retrospective multi-center on gene sequences	DST	1839 isolates	United Kingdom	8 drugs and MDR-TB	ML	AUC 91–100%, Sen 84–97%, Spe 90–98%
[74]	Deelder et al.	Retrospective multi-center on gene sequences	DST	16,688 isolates	Not available	14 drugs and MDR-TB	ML	Acc 73.4–97.5%, Sen 0–92.8%, Spe 75.6–100%
[75]	Chen et al.	Retrospective multi-center on gene sequences	DST	4393 isolates	ReSeqTB Knowledgebase	10 drugs	WDNN and ML	AUC 0.937, Sen 87.9%, Spe 92.7% for the first-line drugs
[76]	Gröschel et al.	Retrospective multi-center on gene sequences	DST	20,408 isolates	NCBI Nucleotide Database	10 drugs	GenTB, WDNN and ML	AUC 0.73–0.96, Sen 57–93%, Spe 78–100%
[77]	Kuang et al.	Retrospective multi-center on gene sequences	DST	10,575 isolates	China, Cameroon, Uganda, etc.	8 drugs	CNN and ML	Acc 89.2–99.2%, Sen 93.4–100%, Spe 48.0–91.7%, F1 score 93.3–99.6%
[78]	Jiang et al.	Retrospective multi-center on gene sequences	DST	12,378 isolates	NCBI-SRA Database	4 drugs	HANN, Attentive neural network	AUC 33.66–99.05%, Sen 67.12–96.31%, Spe 92.52–98.84%

## Data Availability

The original contributions presented in the study are included in the article.
